# High-Resolution SNP/CGH Microarrays Reveal the Accumulation of Loss of Heterozygosity in Commonly Used *Candida albicans* Strains

**DOI:** 10.1534/g3.111.000885

**Published:** 2011-12-01

**Authors:** Darren Abbey, Meleah Hickman, David Gresham, Judith Berman

**Affiliations:** * Department of Genetics, Cell Biology and Development and; § Department of Microbiology, University of Minnesota, Minneapolis, Minnesota 55455, and; † Center for Genomics and Systems Biology; ‡ Department of Biology, New York University, New York, New York 10003

**Keywords:** comparative genome hybridization, single nucleotide polymorphisms, loss of heterozygosity, aneuploidy, genome profiling, haplotype mapping

## Abstract

Phenotypic diversity can arise rapidly through loss of heterozygosity (LOH) or by the acquisition of copy number variations (CNV) spanning whole chromosomes or shorter contiguous chromosome segments. In *Candida albicans,* a heterozygous diploid yeast pathogen with no known meiotic cycle, homozygosis and aneuploidy alter clinical characteristics, including drug resistance. Here, we developed a high-resolution microarray that simultaneously detects ∼39,000 single nucleotide polymorphism (SNP) alleles and ∼20,000 copy number variation loci across the *C. albicans* genome. An important feature of the array analysis is a computational pipeline that determines SNP allele ratios based upon chromosome copy number. Using the array and analysis tools, we constructed a haplotype map (hapmap) of strain SC5314 to assign SNP alleles to specific homologs, and we used it to follow the acquisition of loss of heterozygosity (LOH) and copy number changes in a series of derived laboratory strains. This high-resolution SNP/CGH microarray and the associated hapmap facilitated the phasing of alleles in lab strains and revealed detrimental genome changes that arose frequently during molecular manipulations of laboratory strains. Furthermore, it provided a useful tool for rapid, high-resolution, and cost-effective characterization of changes in allele diversity as well as changes in chromosome copy number in new *C. albicans* isolates.

Genome changes, such as loss of heterozygosity (LOH) or copy number variations (CNV), result in rapid and dramatic large-effect changes in phenotypic diversity. To understand relationships between genotype and phenotype, one must determine the types of changes that arose within clonal subpopulations. This includes measuring gene copy number by array comparative genome hybridization (aCGH) (Hughes *et al.* 2000) and determining allele frequencies by using single nucleotide polymorphism–restriction fragment length polymorphism (SNP-RFLP) analysis (Forche *et al.* 2009b) or using hybridization to microarrays that can distinguish SNP alleles by their differential hybridization properties (Forche *et al.* 2005; Gresham *et al.* 2010).

We use the heterozygous diploid yeast, *C. albicans,* as a model system to study genome dynamics. *C. albicans* is a harmless commensal of the gastrointestinal, genitourinary tract and skin; in hosts with immune deficiencies, it can become a mucosal and/or systemic pathogen. Resistance to fluconazole, the most commonly used antifungal drug, can appear rapidly (Marr *et al.* 1998; Marr *et al.* 1997) and poses a serious challenge to the successful treatment of candidal infections (Arendrup *et al.* 2008; Brion *et al.* 2007; Ruping *et al.* 2008; Sun and Singh 2010). Point mutations, homozygosis of specific resistant alleles, and aneuploidies can give rise to fluconazole resistance [reviewed in Niimi *et al.* (2010)].

Homozygosis of alleles through LOH occurs much more frequently (∼10^−6^ generations) than point mutations (∼10^−9^ generations) (Lang and Murray 2008). LOH events arise through recombination, repair, and/or chromosome segregation mechanisms (Andersen *et al.* 2008). In *C. albicans*, rates of LOH increase when cells are exposed to the stress of growth in the host (Forche *et al.* 2009a) or to physiologically relevant stresses *in vitro* (Forche *et al.* 2011). Despite the fact that LOH causes a decrease in allelic diversity at the individual cell level, it facilitates adaptation by revealing recessive alleles that might provide a selective advantage under stress conditions. For example, homozygosis of hyperactive alleles of several genes (*e.g.* *ERG11*, *TAC1*, and *MRR1)* significantly enhances the ability of *C. albicans* to grow in the presence of fluconazole (Coste *et al.* 2007; Morschhauser *et al.* 2007; White *et al.* 2002). Notably, adaptive gene combinations are predicted to form more frequently when recombination (and thus LOH) rates are higher under stress and lower in the absence of stress (Hadany and Beker 2003).

*C. albicans* laboratory strains are primarily derived from clinical isolate SC5314 (Gillum *et al.* 1984). As the first *C. albicans* isolate with a complete genome sequence (Jones *et al.* 2004), it is the reference strain used to assemble genome sequences of other isolates (Butler *et al.* 2009). It includes, on average, one SNP every 270 bp distributed through ∼81% of the genome. Approximately 19% of the genome, primarily long telomere-proximal domains, is composed of long tracts of homozygosity (Butler *et al.* 2009; Jones *et al.* 2004).

Strain construction in *C. albicans* often incurs unintended alterations in chromosome copy number. A major breakthrough in *C. albicans* genetics was the construction of strain CAI-4, which lacks both copies of the counter-selectable marker *URA3.* However, most CAI-4 isolates carry at least one aneuploid chromosome (Chr1 and/or Chr2 trisomy) (Chen *et al.* 2004; Selmecki *et al.* 2005). The RM series of strains (Alonso-Monge *et al.* 2003), in which both alleles of *HIS1* were sequentially deleted, underwent a deletion of the terminal ∼40 kb of Chr5R distal to the *HIS1* locus (Selmecki *et al.* 2005). Strains that no longer carry these aneuploidies were derived from RM1000 #2, a euploid strain that is homozygous for the distal ∼40 kb of Chr5R (Selmecki *et al.* 2005).

Molecular genetics manipulations, including transformation with DNA and strong counterselection for the excision of markers (*e.g.* *URA3*), changes levels of aneuploidy in laboratory strains (Bouchonville *et al.* 2009). Furthermore, in *C. albicans*, aneuploidy is found in clinical strains: 50% of fluconazole-resistant strains carry at least one aneuploid chromosome. Furthermore, aneuploidy can provide a strong selective advantage with clinical consequences: isochromosome (5L) arose in several different clinical and *in vitro* evolution isolates and confers drug resistance through increased numbers of two genes on Chr5L (Selmecki *et al.* 2006, 2008, 2009). Thus, aneuploidy appears to be a rapid and efficient mechanism used by *C. albicans* to generate a specific selective advantage in the face of strong selective pressure.

To follow large-effect genome changes such as LOH and aneuploidy, it is critically important to efficiently detect genomic changes at high resolution. SNP-RFLP and PCR-based methods follow LOH using a small number of SNP markers on each chromosome arm (Arbour *et al.* 2009; Forche *et al.* 2011, 2009b). In previous work, we established a hapmap with 150-170 markers linked specific alleles to chromosomal homologs and identified some LOH events in strains that underwent the parasexual cycle or that were passaged through a mammalian host (Forche *et al.* 2008, 2009a).

Here, we constructed a microarray that combines ∼39,000 SNP alleles (2 SNP alleles/locus) plus ∼20,000 CGH (non-SNP) loci to simultaneously assess LOH and CNV events in *C. albicans* using commercially available microarrays. Use of this SNP/CGH array, together with strains carrying homozygous or trisomic heterozygous chromosomes, allowed us to assemble a hapmap that distinguishes alleles on each homolog of all eight *C. albicans* chromosomes. Using the SNP/CGH array, we first determined copy number as a function of chromosome position and then used that information to analyze allele ratios across the genome in commonly used *C. albicans* laboratory strains. Importantly, we identified LOH events that accumulated sequentially within this series of strains. It appears that, like aneuploidy, increasing degrees of homozygosity incur small, but detectable, fitness costs.

## Materials and Methods

### Strains and growth media

**Figure 1:**
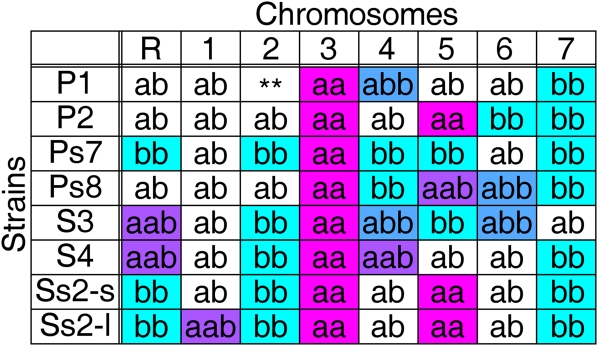
Homolog genotypes of strains used for hapmap construction. Strains derived from a parasexual cross (Forche *et al.* 2008) contained homozygous (“aa” or “bb”) homologs for most or all of ChrR and Chr2–Chr7 and heterozygous trisomies (“aab” or “abb”) as indicated. Chromosomes indicated with “aab“ or “abb“ were trisomic; chromosomes designated “ab” were heterozygous along the entire length of the chromosome. Chromosomes indicated in color were informative for hapmap construction. Chr2 in strain P1 (**) was homozygous for each homolog along different sections of the chromosome. Color schemes for allelic fractions are consistent in Figures 1–4.

*C. albicans* strains used in this study are listed in supporting information, Table S1. We used strains previously shown to be homozygous for one or more chromosomes and/or carrying trisomies (Forche *et al.* 2008; Legrand *et al.* 2008) (Figure 1 and Figure S1, A–H). All strains were grown in rich medium (YPAD) (Guthrie and Fink 1991) at 30° with shaking.

### SNP/CGH microarray design

All probes were designed to be 19 to 33 nt long with melting points (Tm) near 55° (Gresham *et al.* 2010). Initially, probes were designed for 43,658 SNP loci and 28,563 non-SNP loci (including regions that do not have high frequencies of SNPs). SC5314 is homozygous for much of ChrRR, Chr3R, and Chr7L as well as for distal portions of Chr1R, Chr2R, Chr3L, Chr5L, and Chr7R (Butler *et al.* 2009; Jones *et al.* 2004); thus we did not include SNP probes from these segments. Probes were eliminated if they had more than one significant alignment (e-value < 0.001) with the reference genome using BLAST analysis (Altschul *et al.* 1997). The acceptable Tm range was reduced to optimize the number of probes to the Agilent 8×60K microarray format (41,616 SNP allele probes (2 probes per SNP locus, representing the 2 SNP alleles) plus 20,363 non-SNP (CGH) probes (1 per locus)). Quality control of the final array design resulted in a usable SNP allele probe count of 39,222. A total of 19,016 SNP loci (97% of usable SNP loci, Table S2) were informative and were mapped to one of the two homologs. A total of 595 SNP loci (3% of the usable SNP loci, Table S3) were not informative and remained unresolved upon analysis of 24 additional strains (data not shown). These loci likely represent sequences found more than once in the genome or short regions of sequence that differed from the published genome sequence.

Analysis of the final microarray data was performed for the two types of probes separately. First, the CGH probe ratios (experimental relative to diploid reference SC5314) were mapped across the genome (Figure 3), providing copy number data. Segmentation of CGH data into regions with a common copy number was done manually or using ChARM (Myers *et al.* 2004). Distribution of ratio data within each region approximated a normal curve, with the position of the peak indicating copy number. Ratios of ∼1.5 (3:2) or ∼0.5 (1:2) were considered trisomic (Figure 3, Chr5 and Chr6) or monosomic, respectively.

**Figure 2:**
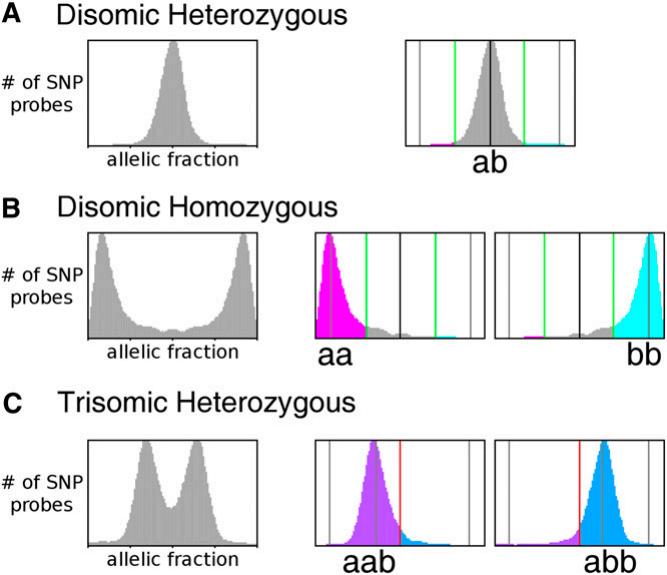
Assignment of SNPs from allele hybridization ratios. Allelic fractions were calculated from SNP array hybridization data for each chromosome in each strain used to construct the hapmap. In each case, unassigned alleles (left panels with only gray data) were plotted with the X-axis as the allelic fraction [b/(a+b)] and the Y-axis as the number of SNP probes, scaled to maximum height of the peaks. (A) For unassigned alleles on heterozygous chromosomes, a = b and b/(a+b) = 0.5 (dark black vertical line); probes that fall between the boundaries (right panel, green lines, values determined as described in *Material and Methods*) are considered heterozygous (gray); probes outside these loci are considered homozygous. (B) For unassigned alleles on homozygous chromosomes, the allelic fractions were close to either 0 or 1 (left panel). For alleles on chromosomes previously designated “aa,” SNP pairs with fractions that fell in the “bb” peak were reassigned to “aa” (magenta, middle panel), and vice versa for “bb” alleles (cyan, right panel) using boundaries that were 50% of the distance between the two peaks in the unassigned data and the heterozygous peak location (green lines). (C) For unassigned alleles on trisomic heterozygous chromosomes, the allelic fractions were close to ∼0.33 or ∼0.67 (“aab” or “abb,” respectively, left panel). SNP pairs with fractions that fell in the “abb” peak were reassigned to “aab” (purple, middle panel), and vice-versa for “aab” alleles (blue, right panel). The boundary between the two regions was set at 50% of the distance between the two peaks (red lines). SNP, single nucleotide polymorphism.

**Figure 3:**
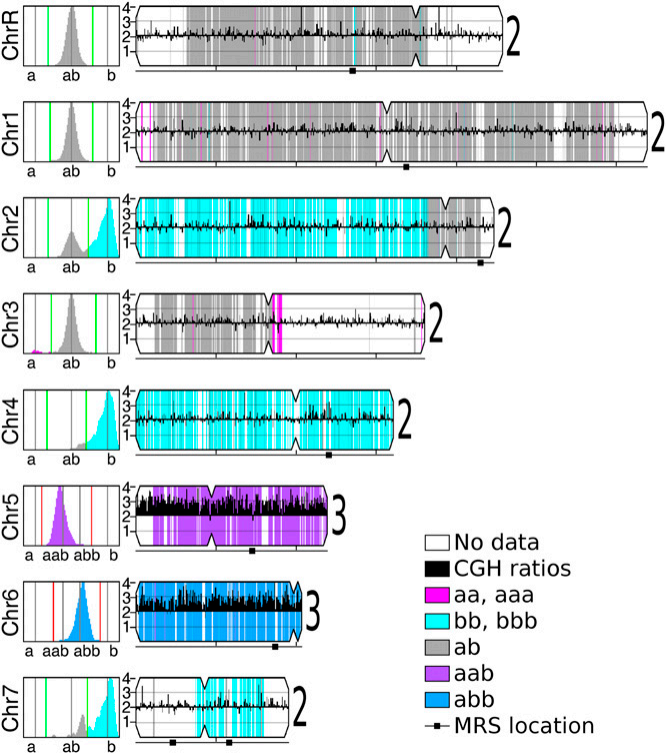
Visualization of SNP/CGH array data. Data for each strain analyzed by SNP/CGH array was visualized as illustrated for strain Ps8 (and in Figure S1 and Figure S2 for all other strains). Each chromosome is illustrated to scale, with its centromere position indicated by an indentation and major repeat sequence (MRS) positions indicted by a black square below the chromosome. CGH data were calculated as ratios, converted to chromosome copy numbers, displayed as black histograms along the length of the chromosome, and summarized with a large numeral to the right of the chromosome. For SNP alleles, allelic fractions for each chromosome are plotted in the left panels. SNP data were calculated and colored as described in Figure 2. Regions that were homozygous in the reference strain or were not informative were not colored. CGH, comparative genome hybridization; SNP, single nucleotide polymorphism.

Second, SNP allele data for experimental strains were normalized to data for the heterozygous diploid reference strain SC5314. Comparison of the hybridization of SNP alleles was used to determine the allelic fractions for each SNP locus. For a disomic heterozygous locus, the theoretical allelic fraction is 0.5 (1:1 ratio), and for a homozygous locus, the theoretical allelic fraction is zero or 1 (0:2 or 2:0 ratio, respectively) (Forche *et al.* 2005). In practice, the disomic homozygous SNP ratios diverged from theoretical values by a small degree (Figure 2B). Allelic fractions were called heterozygous (“ab”) or homozygous (“aa” or “bb”) if they were within one standard deviation of the relevant peak (Figure 2, A *vs.* B). These calls were then mapped as a function of genome position and used to determine the fill color behind the CGH figures. Homozygous regions were displayed as magenta (“aa”) or cyan (“bb”) (Figure 3, Chr2, Chr3, Chr4, and Chr7). Disomic heterozygous regions were displayed as gray (“ab”). For heterozygous chromosomes that were determined to be trisomic by CGH analysis, the theoretical allelic fractions are 0.33 or 0.67 (1:2 or 2:1 ratios, respectively). In practice, the allelic fractions diverged from theoretical values by a small degree (Figure 2C) but were found at the expected 0.33 and 0.67 between the homozygous SNP ratios. Allelic fractions were called as heterozygous (“aab” or “abb”) if they were within one standard deviation of the relevant peak (Figure 2C). These calls were then mapped as a function of genome position and used to determine the fill color behind the CGH figures (Figure 3, Chr5 and Chr6). Trisomic heterozygous regions were displayed as purple (“aab”) or blue (“abb”). Chromosome segments with no known SNPs and/or no SNP probe data remain uncolored.

### DNA isolation, labeling, and microarray hybridization

Genomic DNA was prepared from overnight cultures and labeled with Cy3 or Cy5 thymidine, using Klenow polymerase described in (Selmecki *et al.* 2005). Labeled DNA concentrate was diluted to 16 µl with deionized water. An amount of 4.5 µl of Agilent 10× blocking agent and 22.5 µl of Agilent 2× hybridization buffer were added. Each mixture of Cy3- and Cy5-labeled DNA was treated according to Agilent standard protocols with incubation for 24 hr at 65°. After incubation, arrays were washed using wash buffers and acetonitrile solutions per Agilent standard protocols.

Microarrays were scanned as 16-bit TIFF images and analyzed using “BlueFuse for Microarrays” (BlueGnome, Cambridge). Data were normalized using Block Lowess method and then analyzed at two levels of stringency (one with no data excluded; the second, data excluded when “Quality less than 1; Confidence less than 0.4; PON Ch1 or Ch2 less than 0.6”). In-house scripts, organized into a data analysis pipeline, were used to load, process, and display the data and are available upon request.

### Fitness assays

Cells were grown at 30° to saturation in liquid YPAD media, diluted 1:1000 in fresh YPAD media and grown for 18 hr at 30° in a microplate reader (Sunrise model, Tecan). OD_600_ was measured every 15 min and used for doubling time calculations. Each experiment included a minimum of six biological replicates for each strain, and each experiment was repeated independently at least twice. Error bars reflect the standard deviation from the mean and *P* values were determined by Student *t*-test (Figure 5).

## Results

### Construction of a SNP/CGH microarray for efficient, high-resolution detection of LOH and CNVs

Detection of LOH is facilitated by accurate genotyping of SNP alleles. Recently, isothermal melting temperature probes were shown to maximize discrimination of SNP alleles (Gresham *et al.* 2010), making them ideal for high-resolution analysis of LOH events. To quickly, efficiently, and simultaneously detect large numbers of LOH and CNV events in *C. albicans*, we designed and constructed a custom microarray including ∼39,000 SNP alleles (2 SNP alleles/locus) plus ∼20,000 CGH (non-SNP) loci (see *Materials and Methods* for details) using the Agilent custom array format, which enables simultaneous analysis of eight sets of 60,000 probes. In initial calibration experiments, we found that the non-SNP oligonucleotides accurately reported chromosome copy number and that the SNP allele probes accurately distinguished chromosomes that were known to be heterozygous (Figure 2A) from those known to be homozygous (Figure 2B) (Forche *et al.* 2008).

To assign all SNP alleles on each chromosome to a specific homolog (“a” or “b”), we used lab strains that are homozygous (“aa” or “bb”) or trisomic heterozygous (“aab” or “abb”) for specific chromosomes (Figure 1) (Forche *et al.* 2008; Legrand *et al.* 2008). A total of 97% (38,032) of the SNP alleles were assigned to a specific homolog. The remaining 3% (1190) remained unmapped, either because the SNP was not present in the strains analyzed or because the strains carried multiple SNPs within the locus.

First, we analyzed the CGH loci to determine copy number of whole chromosomes. Disomic (∼1:1 ratio) and trisomic (∼2:1 ratio) chromosomes were readily distinguishable. We then assigned the SNP alleles to a given homolog. SNP allele identity (initially arbitrary) was reassigned to the appropriate homolog using data from disomic homozygous (“aa” and “bb”) and/or trisomic (“aab” and “abb”) chromosomes. For disomic homozygous chromosomes, allelic fractions close to 0 or 1 were generally observed, although there was slight variation with each array experiment (Figure 2B). The threshold was set at 50% of the distance between the homozygous and heterozygous values (Figure 2, A and B, green lines). For chromosomes previously identified as homozygous “bb,” the SNP alleles were reassigned to “bb” if their ratio fell in the “aa” region, and vice versa. For Chr1, no disomic homozygous strain was available. Thus, a strain trisomic and heterozygous for Chr1 (Ss2-l, Figure 1) was analyzed with the allelic fractions shifted (0.33 or 0.67) (Figure 2C), and the threshold was set at 50% of the distance between the two peaks (Figure 2C, red lines). SNP alleles were assigned to a homolog using similar logic to that described above.

SNP/CGH array data collected from eight parasexual progeny isolates (Figure 1) were used to assign SNP alleles (Figure 2). For each strain, data were plotted as a function of chromosome position, and regions of the chromosomes that were informative were shaded to indicate their allelic content (Figure 3 and Figure S1; see *Materials and Methods*).

### Analysis of a series of related laboratory strains

**Figure 4:**
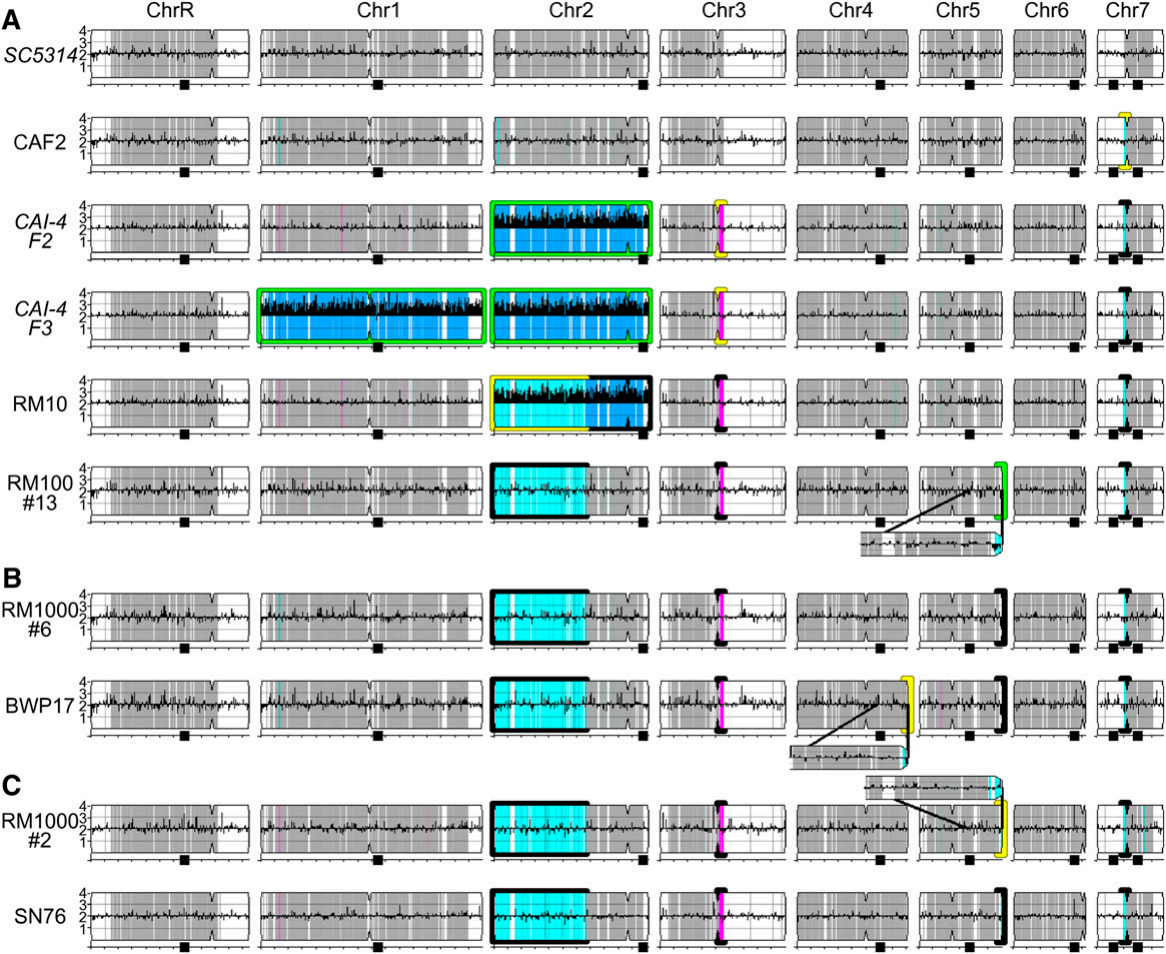
Genome changes acquired during laboratory strain construction. Summary of SNP/CGH analyses of laboratory strains derived from SC5314 with chromosome copy number illustrated as black histograms and homolog identity colored as in Figure 3. Each strain is illustrated horizontally. Strain construction steps proceed from top to bottom. RM100#13 (bottom of A) was the parent for strain lineages in B and C. Genome changes with successive transformation and counterselection steps are enlarged and/or highlighted: new aneuploidies are highlighted with green borders; new LOH events are highlighted with yellow borders. Genome changes that were maintained in subsequent strains are highlighted with black borders. Subfigures for strains with italicized names were simulated from available data and SNP/CGH analysis of additional CAI-4-derived strains. CGH, comparative genome hybridization; LOH, loss of heterozygosity; SNP, single nucleotide polymorphism.

Hapmaps are primarily useful for the study of strains that are closely related to one another. The degree to which *C. albicans* lab strains undergo recombination during transformation and subsequent selection steps has not been determined. Thus, we analyzed the multiply marked strains BWP17 (Wilson *et al.* 1999) and SN76 (Noble and Johnson 2005), as well as several intermediate strains used in their construction from reference strain SC5314. All strains ultimately were constructed from CAI-4 (Figure 4), which lacks both copies of the counterselectable marker *URA3*. CAF2 (Fonzi and Irwin 1993), an intermediate in the construction of CAI-4, is a *ura3∆/URA3* strain derived from SC5314 by transformation (Fonzi and Irwin 1993).

Our analysis of LOH and CNVs in related strains revealed the history of changes that arose during strain construction. In brief, approximately 20% of the SC5314 genome is homozygous (Figure 4A) (Butler *et al.* 2009; Jones *et al.* 2004). In CAF2, SNP/CGH analysis detected an additional 50 kb LOH on Chr7L, which extended the preexisting region of homozygosity (Figure 4A). In CAI-4, an additional region of Chr3R (64 kb) was homozygosed (Figure 4A). In both CAF2 and CAI-4, the LOH event occurred within 12–25 kb of the centromere. Both of these LOH events are found in all derivatives of CAI-4. Furthermore, as noted previously (Selmecki *et al.* 2005), some CAI-4 isolates (*e.g.* CAI-4 F2, Figure 4A) have one trisomy (Chr2), whereas others (*e.g.* CAI-4 F3, Figure 4A) have two (Chr1 and Chr2).

RM10 (*his1∆/HIS1, ura3∆∆*) was derived from a CAI-4 isolate by transformation and subsequent FOA selection; it acquired an LOH event that spans most of the left arm of trisomic Chr2 (Figure 4A, 1330 kb). RM100 #13 (*his1∆/his1∆::URA3*, *ura3∆∆)* lost one copy of Chr2, retaining the Chr2R “a” and “b” alleles, and it also lost a portion (∼40 kb) of the Chr5R “a” homolog distal to *HIS1* (Figure 4A) (Selmecki *et al.* 2005). These changes were retained in strains RM1000 #6 (Figure 4B, *his1∆∆, ura3∆∆*) and BWP17 (Figure 4B, *arg4∆∆*, *his1∆∆*, *ura3∆∆).* The Chr5R distal, hemizygous region became homozygous disomic (“b” alleles) in transformant RM1000 #2 (Figure 4C, *ura3∆∆*, *his1∆∆*) (Selmecki *et al.* 2005), and this was retained in its derivative SN76 (Figure 4C, *ura3∆∆*, *his1∆∆*, *arg4∆∆*). Both BWP17 and SN76 underwent two transformations and two counterselections on 5-FOA to delete both copies of *ARG4.* BWP17 acquired an additional LOH on the terminal 30 kb of Chr4R during one of these stages, whereas the SN76 genotype is indistinguishable from that of RM1000 #2. Taken together, these results indicate that LOH, like aneuploidy, often arises during strain constructions involving DNA transformation. These LOH events generate lab strains that are more homozygous than SC5314.

### Fitness consequences of LOH and aneuploidy

**Figure 5:**
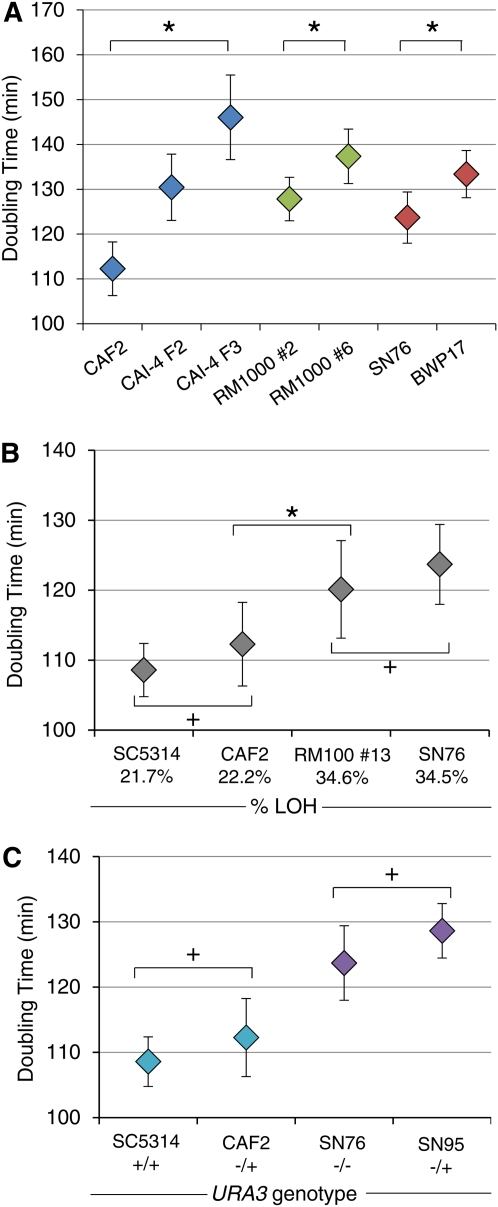
*In vitro* growth of lab strains with different degrees of ploidy or LOH or with different *URA3* copy number. (A) Deviations from diploidy correlate with increased doubling times. Doubling times (min) were measured for strains with similar degrees of LOH and different degrees whole-genome ploidy. Blue: CAF2 (2N), CAI-4 F2 [2.16N (2N+Chr2)] and CAI-4 F3 [2.38 (2N + (Chr1 + Chr2))] Green: RM1000 #2 (2N) and RM1000 #6 (1.997N) have identical genome content but are homozygous or hemizygous, respectively, for the terminal ∼40 kb of Chr5R. Red: SN76 (2N) and BWP17 (1.997N), like the RM1000 strains, are homozygous and hemizygous for the tip of Chr5R; in addition, BWP17 acquired a small (30 kb) LOH on Chr4R. *Within each group, all strains are significantly different from each other (*P* < 0.05, Student *t*-test). (B) Increases in percentage of the genome that is homozygous (as indicated on X-axis) correlate with increased doubling times. SC5314 and CAF2 are not different from each other (+) but are significantly different from RM100 #13 and SN76 (*P < 0.05, Student *t*-test). (C) *URA3* status does not affect *in vitro* doubling times in medium containing uridine. Pairs of strains (teal and purple) with similar ploidies and similar degrees of LOH that differ by one copy of *URA3*: SC5314 *(URA3/URA3)* *vs.* CAF2 *(URA3/ura3∆),* or SN76 (*ura3∆/ura3∆)* *vs.* SN95 *(URA3/ura3)*) were not significant different in doubling time relative to one another (+). LOH, loss ofheterozygosity.

Ultimately, accumulated genomic changes have the potential to affect the cellular fitness of an organism. Fitness in asexual microorganisms such as *C. albicans* is often determined by population doubling time, although the maximum number of doublings in a population also plays an important role (Addinall *et al.* 2011). In yeasts, aneuploidy often causes reduced fitness [reviewed in Torres *et al.* (2008)], although specific aneuploidies can also confer fitness advantages under selective as well as nonselective conditions (Dunham *et al.* 2002; Gresham *et al.* 2008; Pavelka *et al.* 2010; Selmecki *et al.* 2006). To distinguish between the effects of aneuploidy and LOH on fitness, we measured the doubling times for SC5314 and its derivatives (Figure 5) under standard laboratory conditions. Sets of strains with similar degrees of LOH and different ploidies, as well as sets of strains with similar ploidies and different degrees of LOH, were compared. Overall, doubling-time measurements mirrored fitness measurements that incorporate the maximum number of doublings in a population (Figure S3).

Strains harboring extra copies of large chromosomes had significantly increased doubling times compared with a euploid strain with a similar degree of LOH (Figure 5A, blue). Even very small changes in ploidy (*e.g.* truncation of 40 kb from Chr5R) resulted in longer doubling times (Figure 5A, green). Importantly, these strains have essentially identical genome content: RM1000 #6 is hemizygous and RM1000 #2 is homozygous for the terminal portion of Chr5R. A similar difference in Chr5R and a similar decrease in fitness distinguished BWP17 from SN76 (Figure 5A, red), although we cannot rule out contributions from the small Chr4R LOH present in BWP17 but not in SN76.

The effects of LOH on fitness are not as well documented, because high-resolution methods to measure LOH have not been widely available. Here, we compared the fitness of strains with similar ploidies and different degrees of LOH [SC5314 and CAF2 (Chr7L); RM100 #13 and SN76 (Chr2L, Chr3R, Chr5R, and Chr7L)] under replete growth conditions that should neutralize the contributions of any auxotrophic markers. Interestingly, larger tracts of homozygosity correlated with increased doubling time (Figure 5B), but small increases in homozygosity did not. It is very likely that this is due to the specific alleles involved in the LOH tracts rather than the size of the LOH tract *per se*.

In *C. albicans*, *ura3∆/ura3∆* strains exhibit altered morphogenesis and reduced virulence (Bain *et al.* 2001; Chen *et al.* 2003; Noble and Johnson 2005). Yet *in vitro* fitness costs have not been determined for *ura3∆/ura3∆* isolates. Comparison of *in vitro* growth of two sets of strains that differ in *URA3* status, yet have similar ploidy (SC5314 and CAF2; SN76 and SN95), revealed no significant change in doubling time (Figure 5C). Thus, under standard lab conditions, using YPAD medium supplemented with uridine, the *URA3* genotype has no significant effect on fitness. In general, aneuploidy and LOH both reduce fitness *in vitro*. Importantly, this is likely due to multiple allele-specific effects in both cases. Furthermore, the degree to which they affect fitness likely differs under different growth conditions.

## Discussion

Long-range chromosomal changes such as LOH and CNVs confer large-effect phenotypic variation. Here we describe microarrays that perform both high-resolution SNP and CGH analysis simultaneously. When used together with strains that are homozygous or trisomic, this technology reveals a high-resolution hapmap that allows homolog phasing of the largely heterozygous *C. albicans* genome. In addition, analysis of lab strain construction history revealed increasing levels of LOH with increasing strain manipulation. We assume that stresses involved in transformation, as well as the stress of growing on counterselection medium, increases the frequencies of LOH as it increases aneuploidy (Bouchonville *et al.* 2009).

Aneuploidy is dynamic and reversible. For example, trisomies can be lost via nondisjunction, restoring the original genotype or resulting in whole-chromosome homozygosis. Recombination between trisomic chromosomes and subsequent chromosome loss can result in large segmental LOH tracts (*e.g.* Chr2 in RM10 and its derivatives, Figure 4). Shorter segmental monosomies (*e.g.* Chr5R terminus in RM100) can be restored to disomy, but the alleles remain homozygous. Thus, while aneuploidies can appear and disappear relatively quickly, they can affect the degree of homozygosity. Thus, aneuploidies can cause transient or permanent genome changes.

If LOH events occur in aneuploid or euploid strains, the loss of allelic diversity cannot be reversed without events involving mating or mutagenesis. Accordingly, lab strains that underwent more manipulations generally have accumulated more LOH events. These LOH events and aneuploidies do not confer a selective advantage under standard laboratory conditions. In contrast, aneuploidies and LOH events found in azole-resistant clinical isolates can provide a clear selective advantage in the presence of the drug due to specific gene contributions (Coste *et al.* 2007; Selmecki *et al.* 2008). This is reminiscent of the situation in *S. cerevisiae*, where aneuploidies are usually detrimental to growth in standard conditions (Pavelka *et al.* 2010; Torres *et al.* 2007) but can provide a selective advantage under some stress conditions (Pavelka *et al.* 2010; Rancati *et al.* 2008) and in some genetic backgrounds (Hughes *et al.* 2000).

Paradoxically, despite the stable accumulation of LOH tracts with sequential exposure to stress, most clinical isolates are largely heterozygous. This implies that parasexual mating between nonsisters must occur at a high enough frequency to restore the observed heterozygosity. Alternatively, selection forces *in vivo* strongly favor the survival of strains with more heterozygosity.

Heat shock, which is used to isolate transformants, clearly promotes aneuploidy (Bouchonville *et al.* 2009; Hilton *et al.* 1985). This may be due to depletion of HSP90 and its cofactor Sgt1, which in *Saccharomyces cerevisiae* is required for assembly and stability of centromere/kinetochore and centrosome complexes (Bansal *et al.* 2009; Zhang *et al.* 2008) and may influence the incidence of aneuploidy in that organism. Elevated temperature, such as that in febrile patients (*e.g.* 39°), also results in increased frequencies of LOH (Forche *et al.* 2011), and thus, the heat shock step in DNA transformations may promote recombination that leads to LOH as well. However, it should be noted that transformation by electroporation also results in increased aneuploidy (Bouchonville *et al.* 2009).

Strong selection pressure applied to identify rare transformants also promotes the appearance of aneuploidy [*e.g.* on 5-FOA, (Wellington and Rustchenko 2005)]. LOH rates also increase under the stress of growth *in vivo* (Forche *et al.* 2009a) and in response to fluconazole or oxidative stress applied *in vitro* (Forche *et al.* 2011).

The effects of large aneuploidies, especially extra copies of Chr1 and Chr2, which increase ploidy by ∼20%, had a more dramatic effect on doubling time and fitness than the most extensive cumulative LOH (Figure 5, BWP17 and SN76, homozygous for almost ∼35% of the genome). However, because SC5314 is already homozygous for ∼20% of the genome, there may be less dynamic range available to detect effects of LOH on fitness. Importantly, the effect of LOH and aneuploidy on fitness is likely to vary, depending upon the specific genes and alleles involved, as well as on the growth conditions being used to measure fitness.

In summary, we designed and demonstrated the utility of a combined SNP/CGH array for high-resolution, rapid, and accurate detection of large-effect genome changes, including both LOH and CNV events. This array, together with its analysis pipeline, is a useful and cost-effective tool for characterizing laboratory as well as clinical strains. Furthermore, this study highlights that whole-genome analysis of engineered mutants should be performed prior to using such mutants in critical experiments. This will facilitate the identification of strains with the desired levels of ploidy and heterozygosity. In addition, the hapmap alleles provided here will greatly facilitate the phasing of SNPs in commonly used laboratory strains. This is essential for optimal appreciation of allele specificity found within next-generation sequencing data. Clearly, once high-throughput sequencing and data analysis becomes affordable and accessible to all researchers, it will obviate the need for array analysis. However, until that time, this technology and the data pipelines for its analysis are important and useful tools for the *Candida albicans* research community.

## Supplementary Material

Supporting Information

Corrigendum
