# Supplementary material for: High-Resolution SNP/CGH Microarrays Reveal the Accumulation of Loss of Heterozygosity in Commonly Used Candida albicans Strains
Source: G3 (Bethesda). 2011 Dec 1;1(7):523–30. doi: 10.1534/g3.111.000885 (PMC3276171; doi:10.1534/g3.111.000885)
Supplement: Supporting Information [file supp_1_7_523__index.html]

Supporting Information 

# High-Resolution SNP/CGH Microarrays Reveal the Accumulation of Loss of Heterozygosity in Commonly Used *Candida albicans* Strains

## Supporting Information for Hickman *et al.*, 2011

**Files in this Data Supplement:**

- Supporting Information - Figures S1-S3 (PDF, 1.1 MB)
- Figure S1 - SNP/CGH of parasexual progeny strains used to construct the *C. albicans* SC5314 hapmap (PDF, 596 KB)
- Figure S2 - SNP/CGH figures of related laboratory strains derived from SC5314 (PDF, 328 KB)
- Figure S3 - Doubling time correlates with strain fitness (PDF, 56 KB)
- Table S1 - Strains used in this study (Microsoft Excel, .xlsx, 12 KB)
- Table S2 - Informative SNPs in the complete Hapmap (Microsoft Excel, .xls, 2.8 MB)
- Table S3 - SNPs on the array that were not informative (Microsoft Excel, .xls, 114 KB)
