# Supplementary material for: High-Resolution SNP/CGH Microarrays Reveal the Accumulation of Loss of Heterozygosity in Commonly Used Candida albicans Strains
Source: G3 (Bethesda). 2011 Dec 1;1(7):523–30. doi: 10.1534/g3.111.000885 (PMC3276171; doi:10.1534/g3.111.000885)
Supplement: Supporting Information [file supp_1.7.523_000885SI.pdf]

A

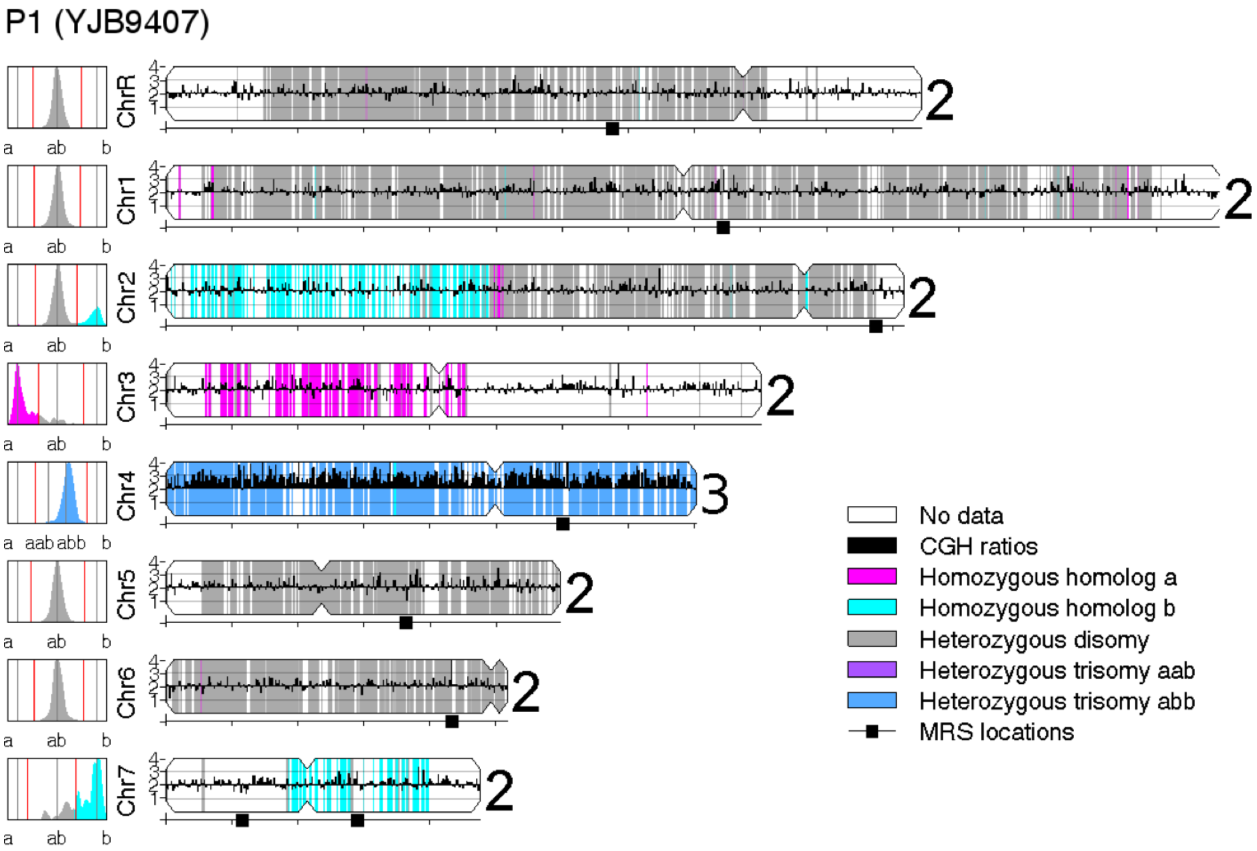

P1 (YJB9407)
ChrR
Chr1
Chr2
Chr3
Chr4
Chr5
Chr6
Chr7
a ab b
a aab abb b
4
3
2
1
2
2
2
2
3
2
2
2
No data
CGH ratios
Homozygous homolog a
Homozygous homolog b
Heterozygous disomy
Heterozygous trisomy aab
Heterozygous trisomy abb
MRS locations

B

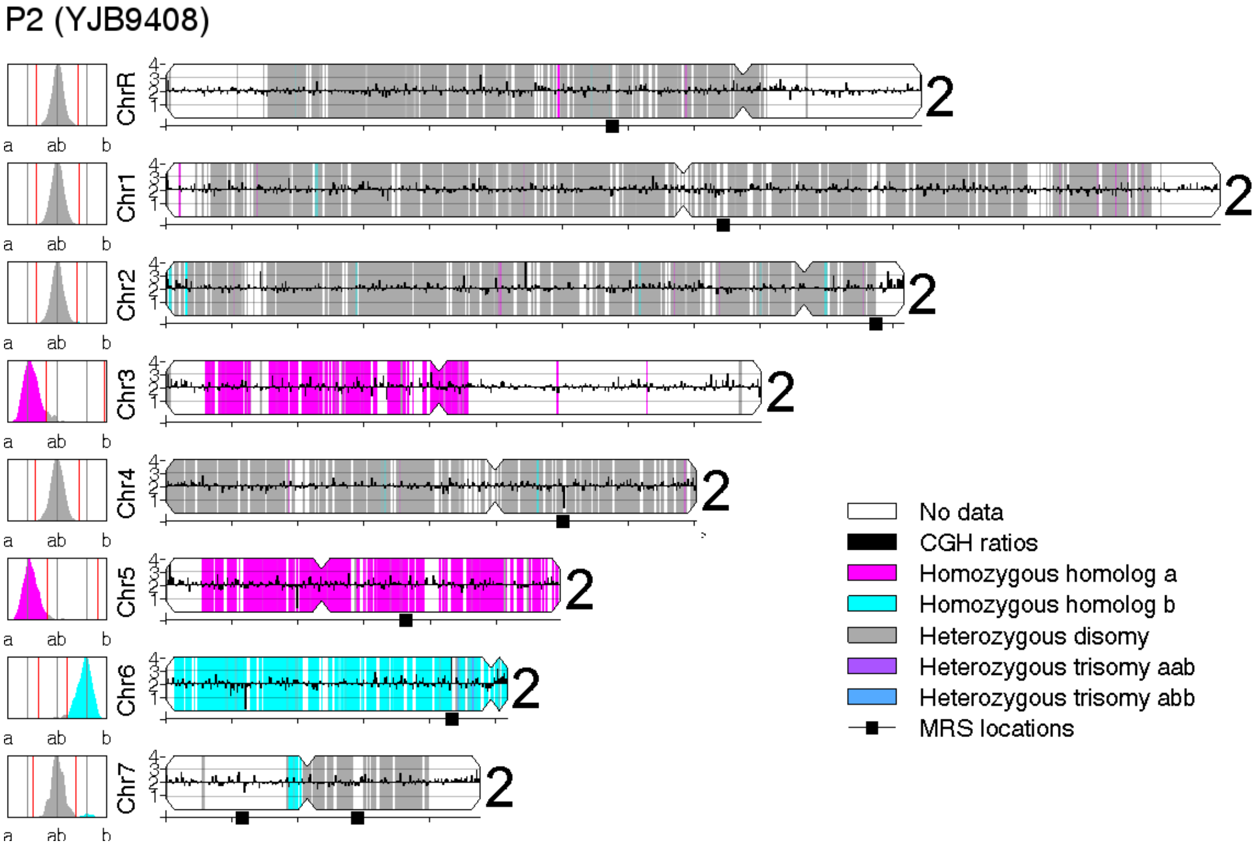
P2 (YJB9408)
ChrR
Chr1
Chr2
Chr3
Chr4
Chr5
Chr6
Chr7
a
ab
b
No data
CGH ratios
Homozygous homolog a
Homozygous homolog b
Heterozygous disomy
Heterozygous trisomy aab
Heterozygous trisomy abb
MRS locations

C

Ps7 (YJB10019)

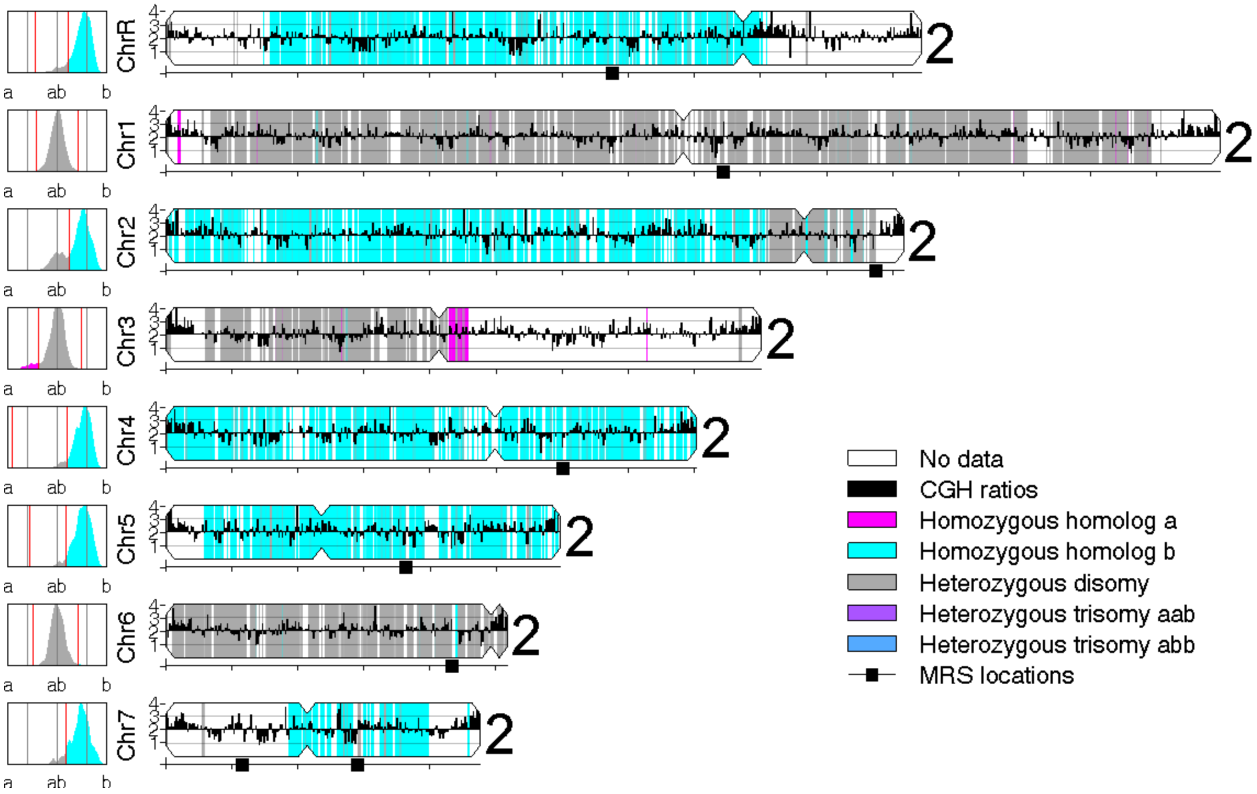

D

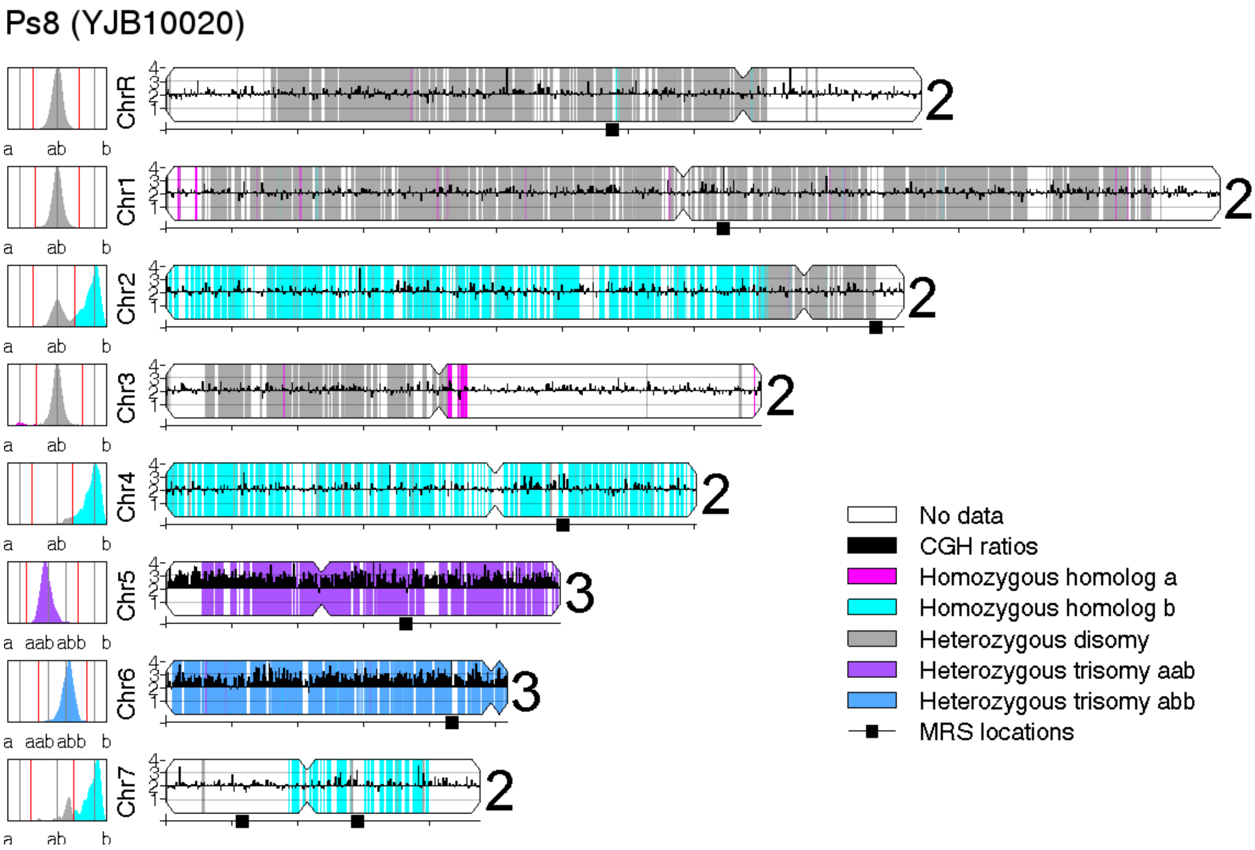
Ps8 (YJB10020)
ChrR
Chr1
Chr2
Chr3
Chr4
Chr5
Chr6
Chr7
a
ab
b
aab
abb
2
2
2
2
2
3
3
2
No data
CGH ratios
Homozygous homolog a
Homozygous homolog b
Heterozygous disomy
Heterozygous trisomy aab
Heterozygous trisomy abb
MRS locations

E

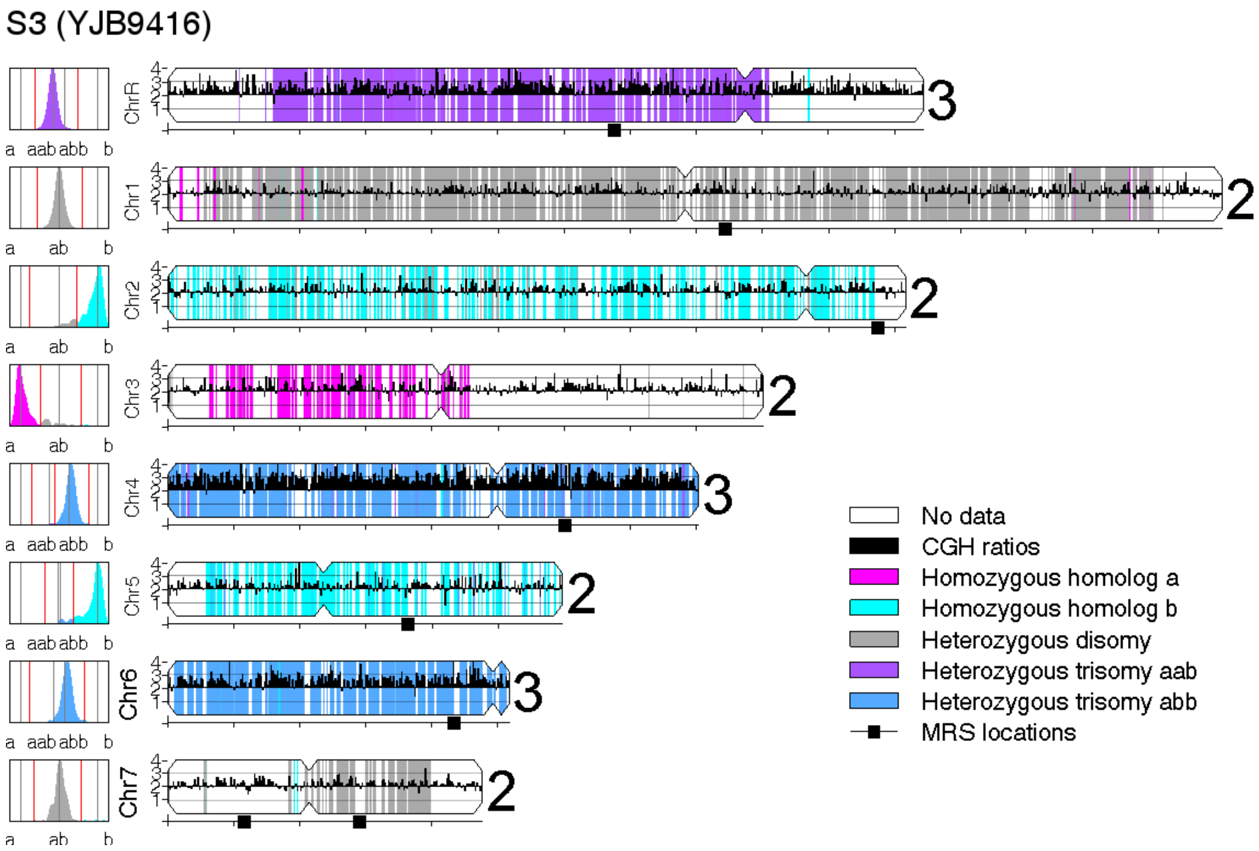

S3 (YJB9416)
ChrR
3
Chr1
2
Chr2
2
Chr3
2
Chr4
3
Chr5
2
Chr6
3
Chr7
2
a aab abb b
a ab b
No data
CGH ratios
Homozygous homolog a
Homozygous homolog b
Heterozygous disomy
Heterozygous trisomy aab
Heterozygous trisomy abb
MRS locations

F

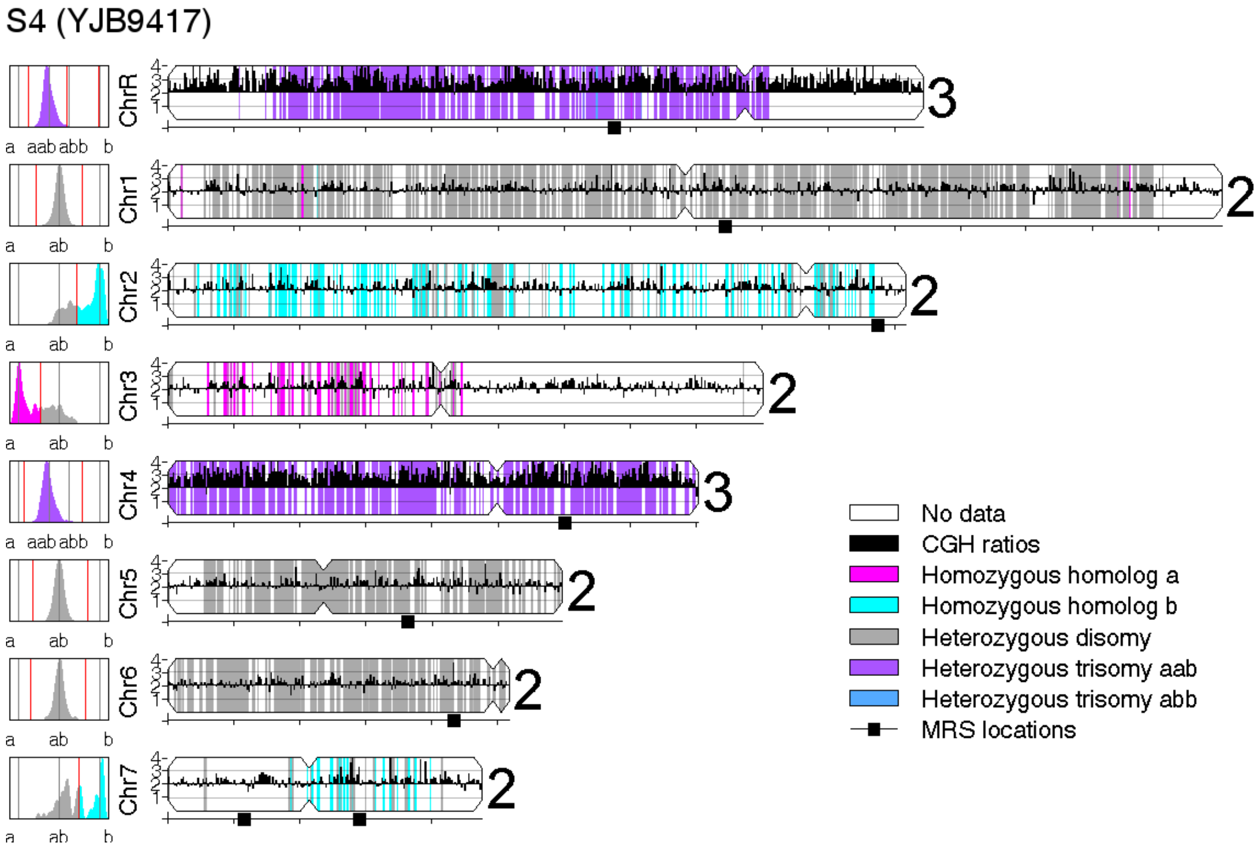
S4 (YJB9417)
ChrR
Chr1
Chr2
Chr3
Chr4
Chr5
Chr6
Chr7
a aab abb b
a ab b
3
2
2
2
3
2
2
2
No data
CGH ratios
Homozygous homolog a
Homozygous homolog b
Heterozygous disomy
Heterozygous trisomy aab
Heterozygous trisomy abb
MRS locations

G

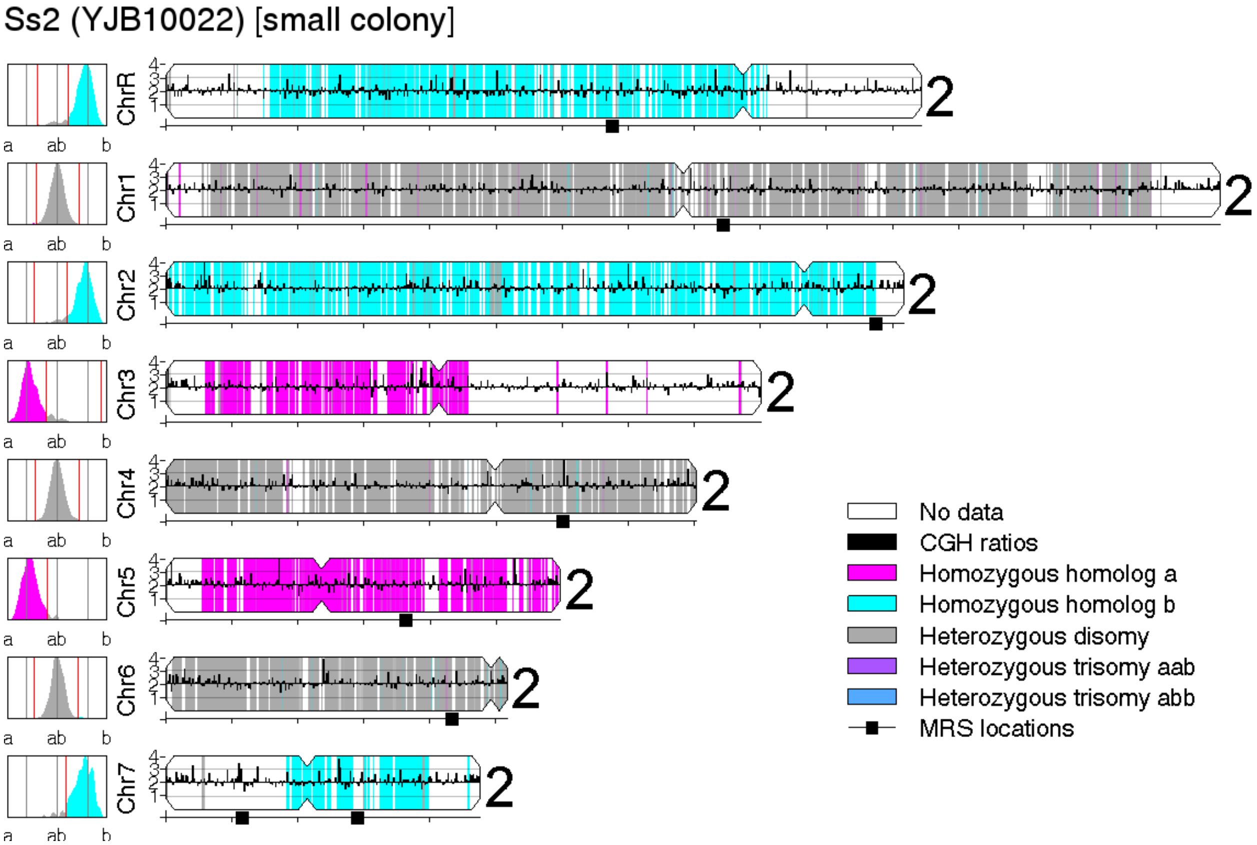
Ss2 (YJB10022) [small colony]
ChrR
Chr1
Chr2
Chr3
Chr4
Chr5
Chr6
Chr7
a
ab
b
2
No data
CGH ratios
Homozygous homolog a
Homozygous homolog b
Heterozygous disomy
Heterozygous trisomy aab
Heterozygous trisomy abb
MRS locations

H

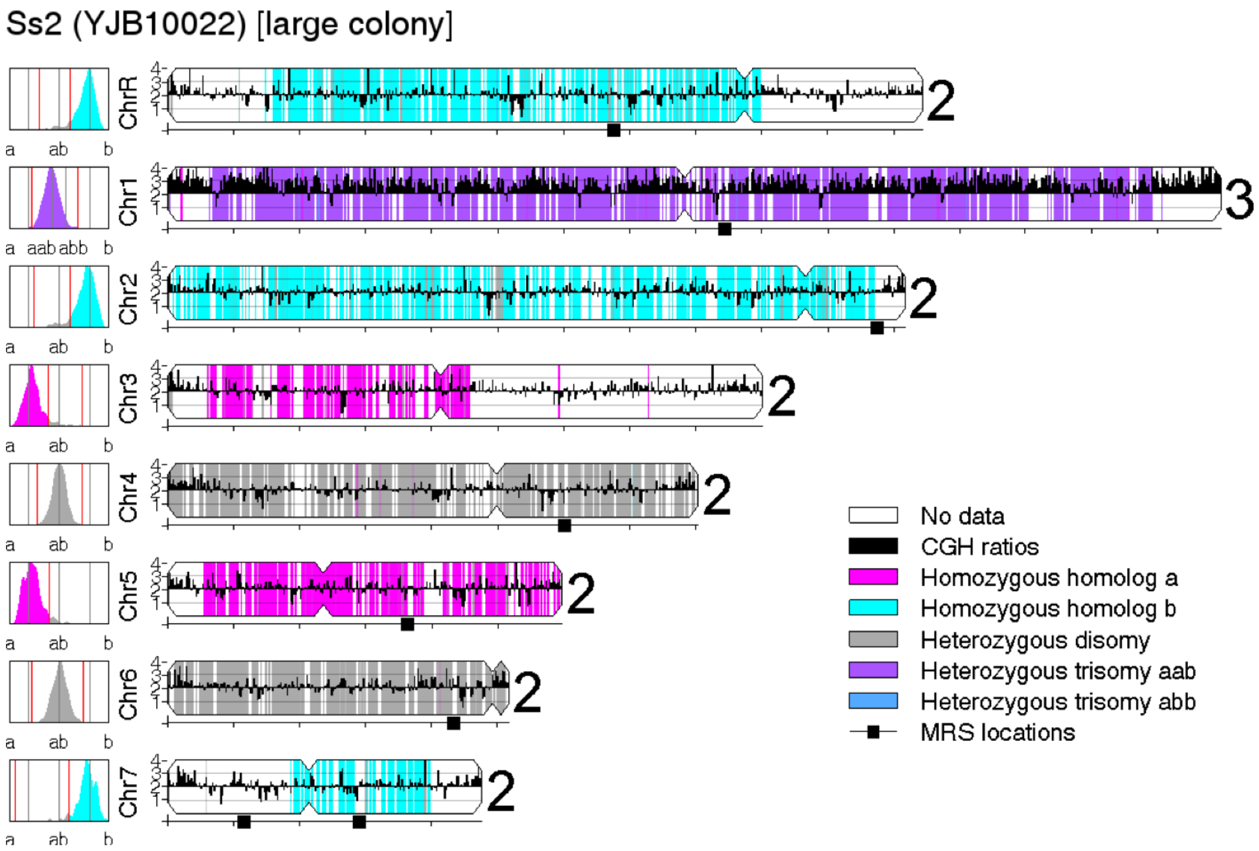

Figure S1 SNP/CGH of parasexual progeny strains used to construct the *C. albicans* SC5314 hapmap. Data for each strain was analyzed by SNP/CGH array and visualized as illustrated in Fig. 3. A) Strain P1 (YJB9407); B) P2 (YJB9408). C) Ps7 (YJB10019). D) Ps8 (YJB10020). E) S3 (YJB9416). F) S4 (YJB9417). G) Ss2 (YJB10022), small colony. H) Ss2 (YJB10022), large colony.

A

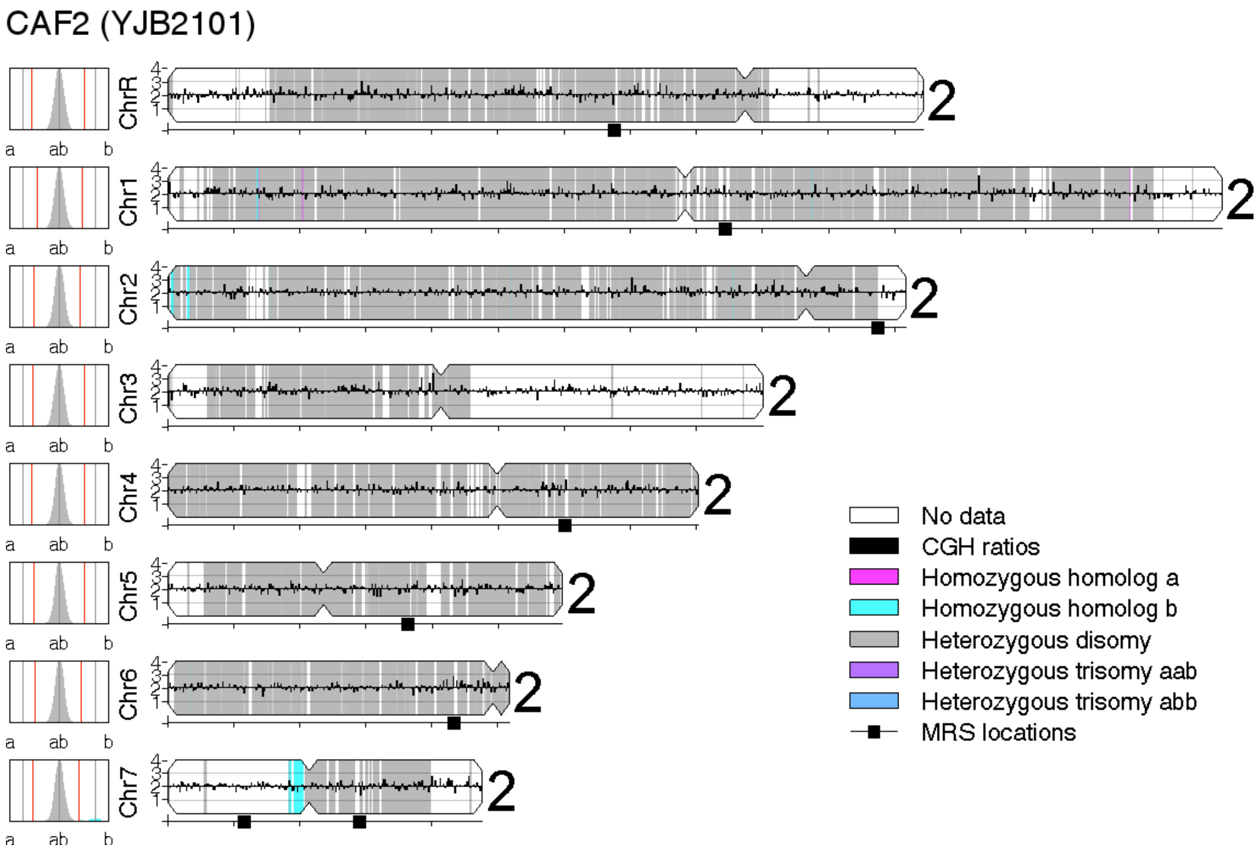
CAF2 (YJB2101)
ChrR
Chr1
Chr2
Chr3
Chr4
Chr5
Chr6
Chr7
a
ab
b
No data
CGH ratios
Homozygous homolog a
Homozygous homolog b
Heterozygous disomy
Heterozygous trisomy aab
Heterozygous trisomy abb
MRS locations

B

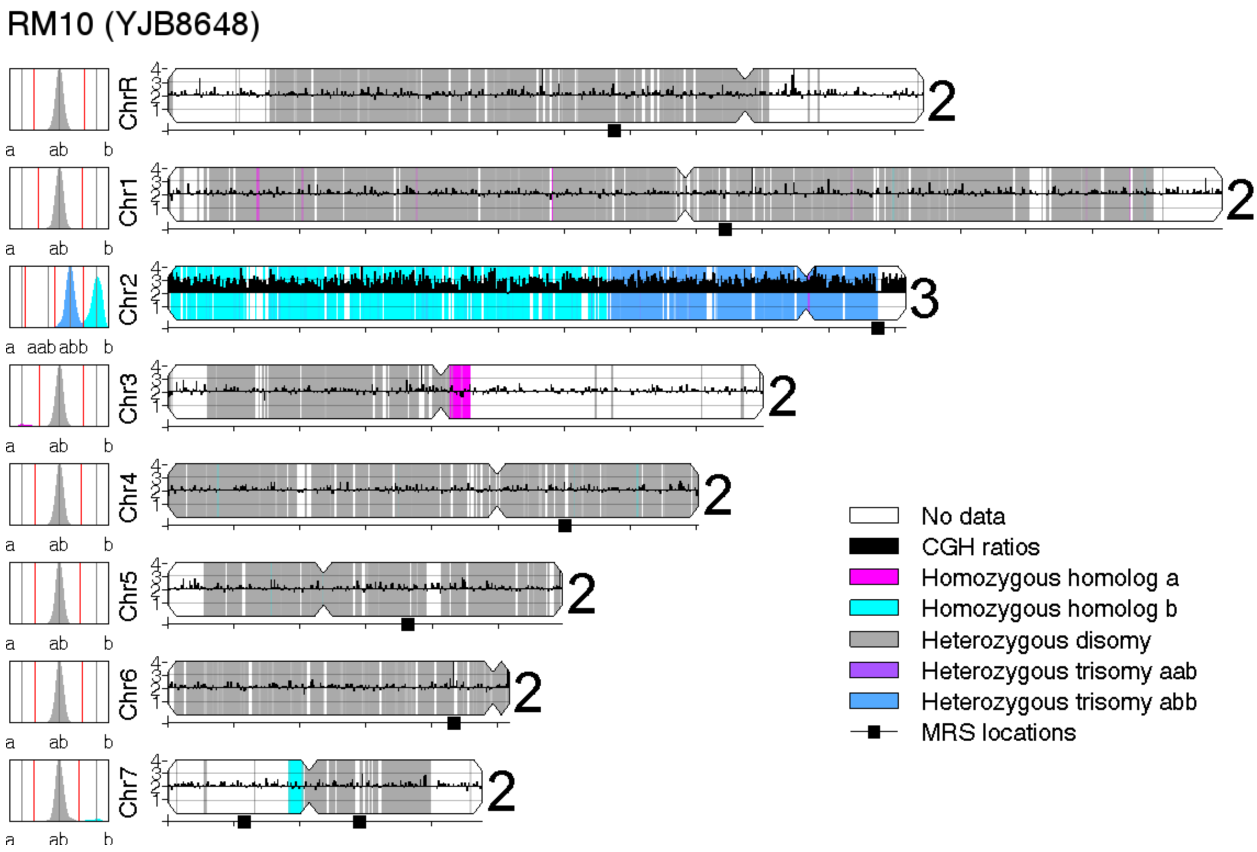
RM10 (YJB8648)
ChrR
2
Chr1
2
Chr2
3
Chr3
2
Chr4
2
Chr5
2
Chr6
2
Chr7
2
a
ab
b
aab
abb
No data
CGH ratios
Homozygous homolog a
Homozygous homolog b
Heterozygous disomy
Heterozygous trisomy aab
Heterozygous trisomy abb
MRS locations

c

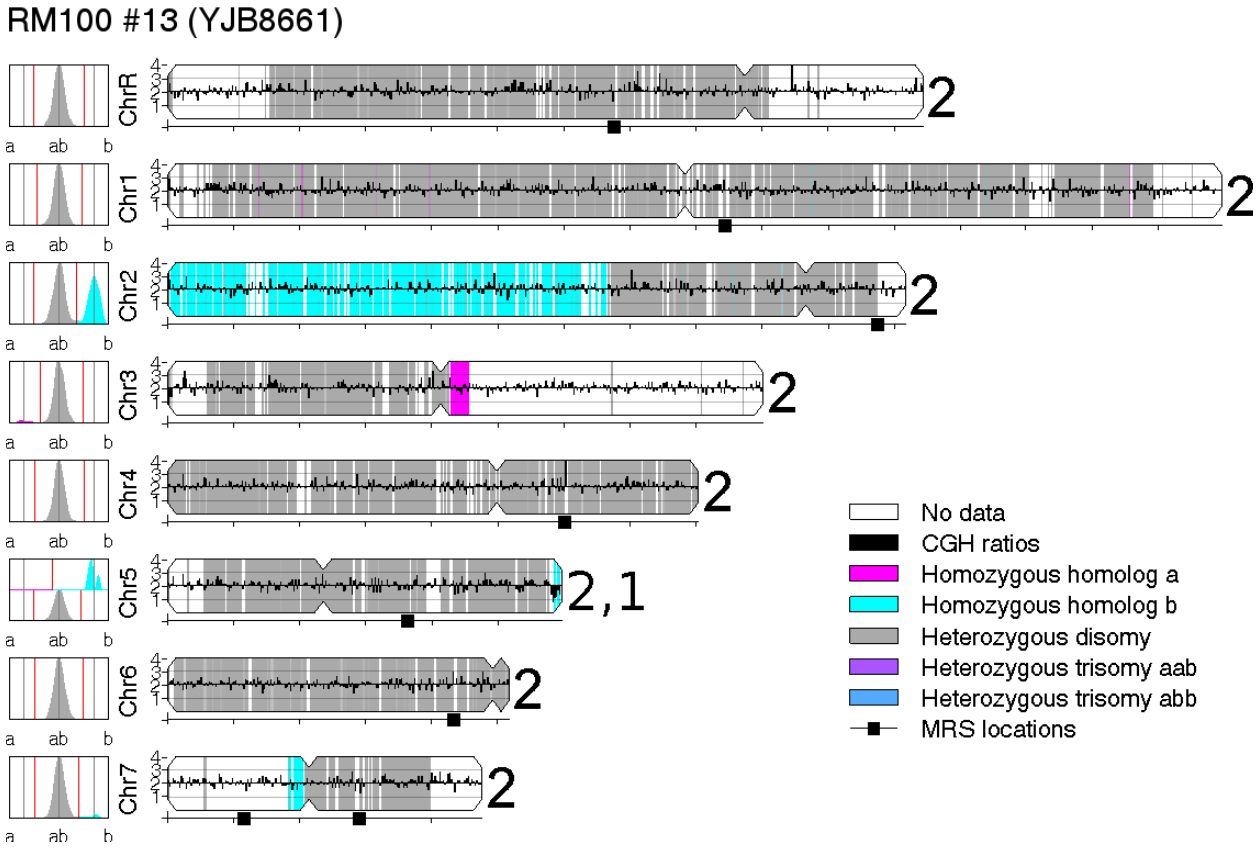

RM100 #13 (YJB8661)
ChrR
Chr1
Chr2
Chr3
Chr4
Chr5
Chr6
Chr7
a
ab
b
2
2,1
No data
CGH ratios
Homozygous homolog a
Homozygous homolog b
Heterozygous disomy
Heterozygous trisomy aab
Heterozygous trisomy abb
MRS locations

D

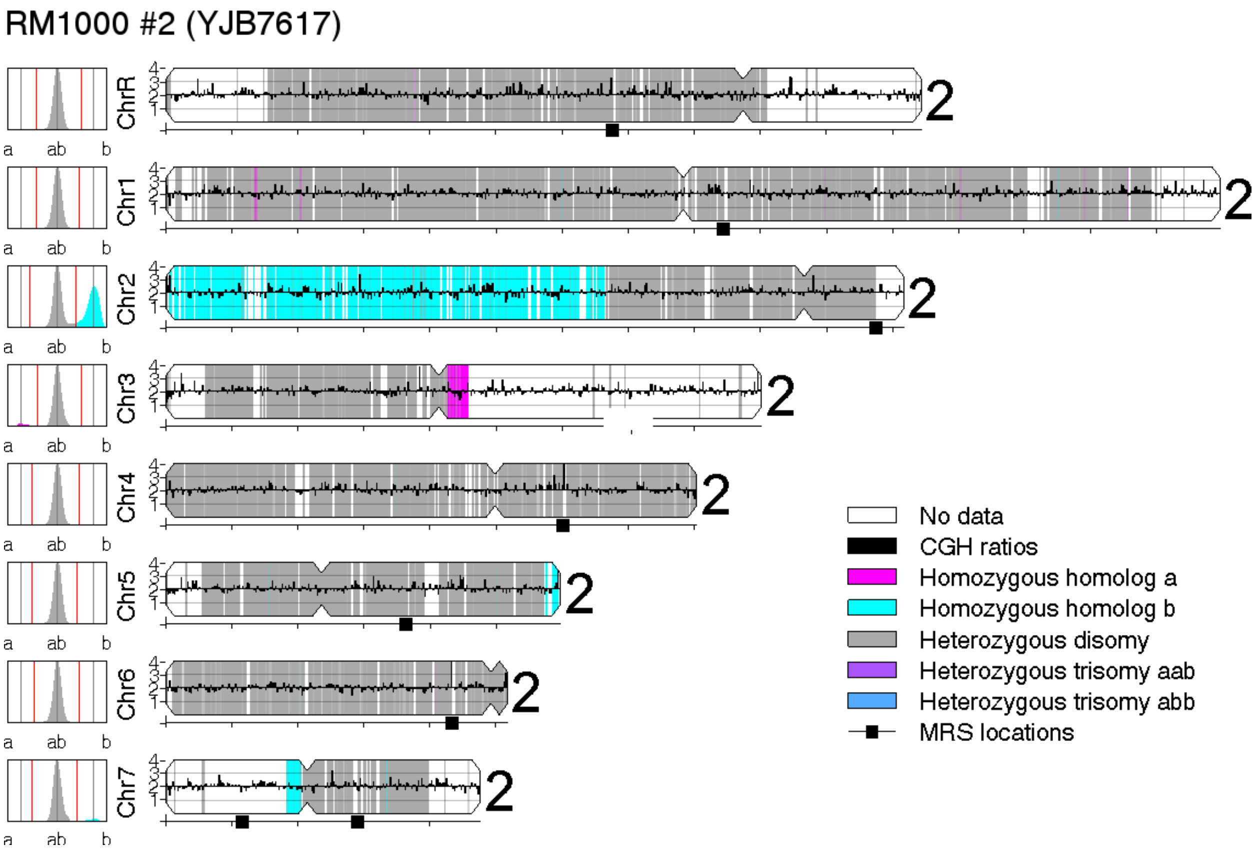
RM1000 #2 (YJB7617)
ChrR
Chr1
Chr2
Chr3
Chr4
Chr5
Chr6
Chr7
a
ab
b
2
No data
CGH ratios
Homozygous homolog a
Homozygous homolog b
Heterozygous disomy
Heterozygous trisomy aab
Heterozygous trisomy abb
MRS locations

E

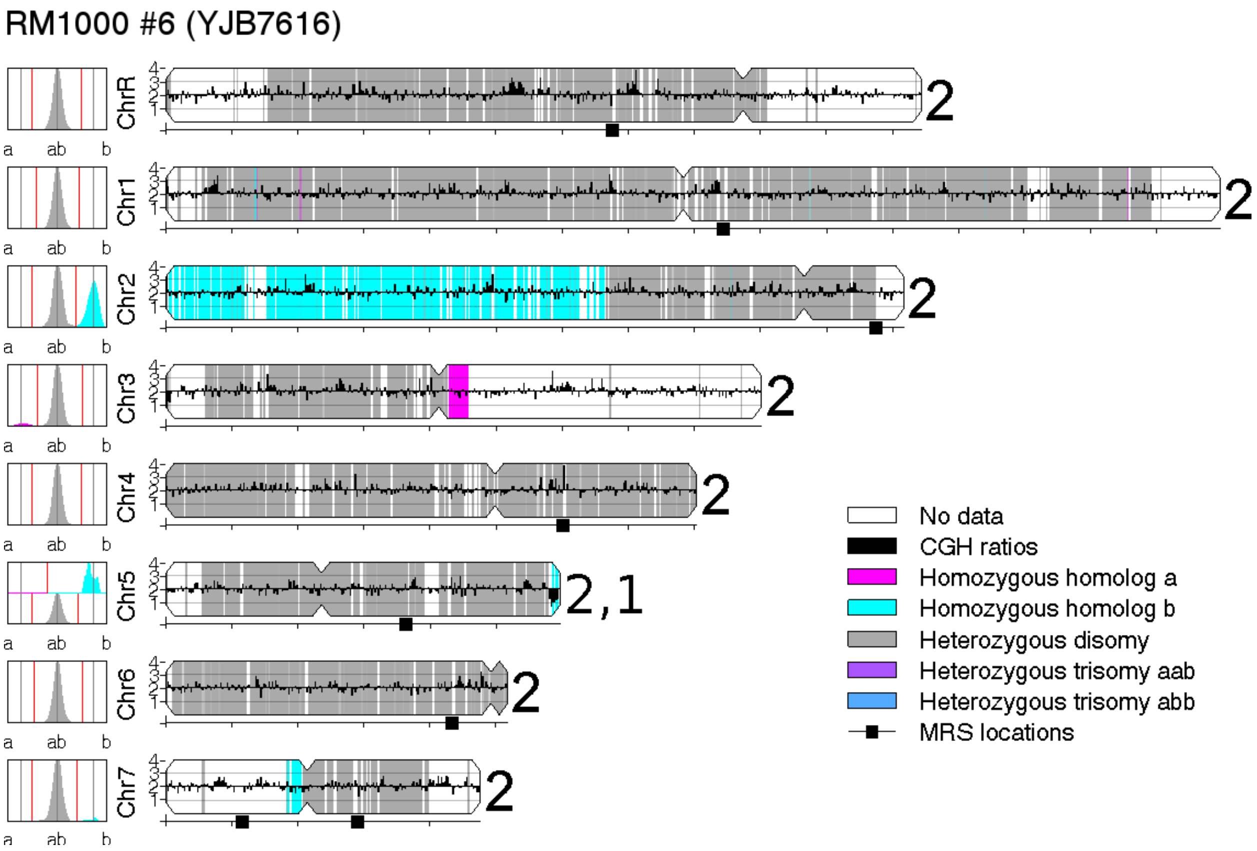
RM1000 #6 (YJB7616)
ChrR
Chr1
Chr2
Chr3
Chr4
Chr5
Chr6
Chr7
a ab b
2
2,1
No data
CGH ratios
Homozygous homolog a
Homozygous homolog b
Heterozygous disomy
Heterozygous trisomy aab
Heterozygous trisomy abb
MRS locations

F

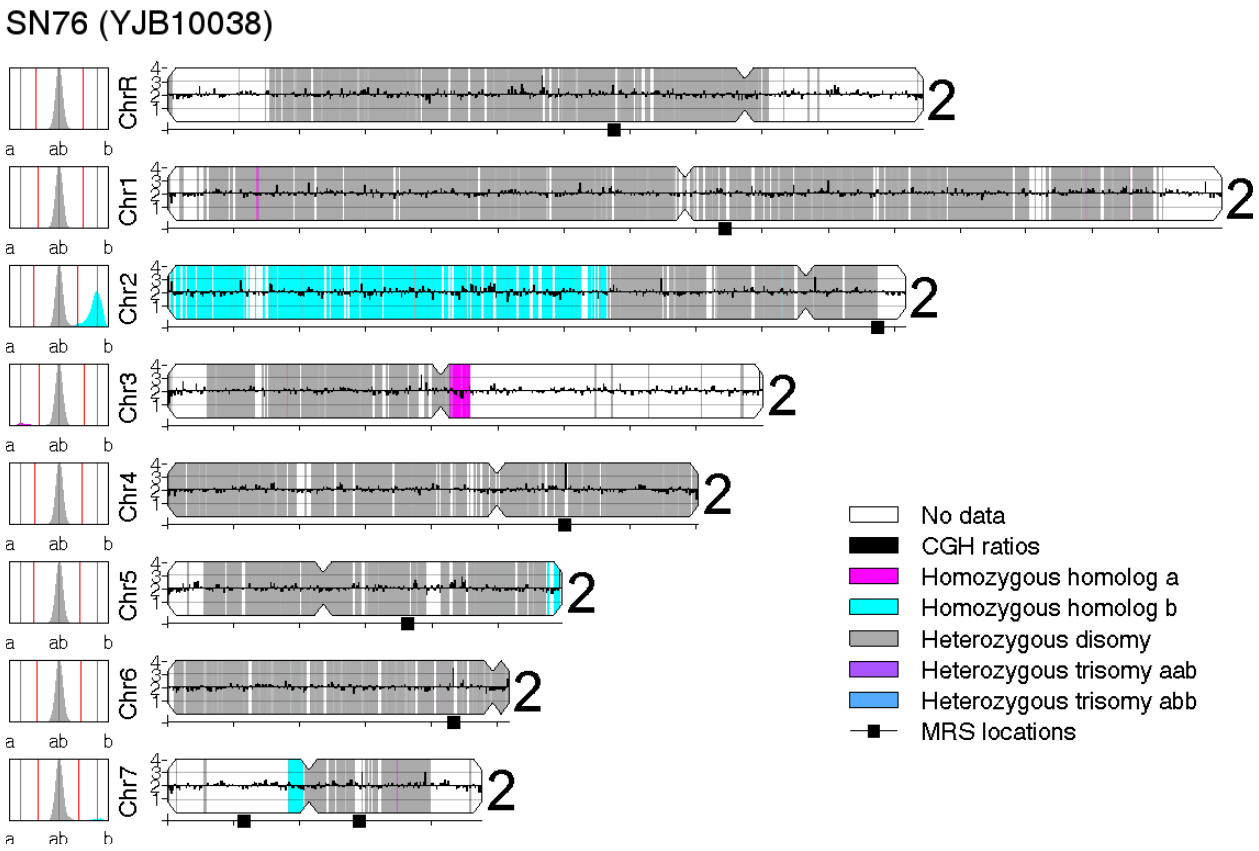
SN76 (YJB10038)
ChrR
Chr1
Chr2
Chr3
Chr4
Chr5
Chr6
Chr7
a
ab
b
No data
CGH ratios
Homozygous homolog a
Homozygous homolog b
Heterozygous disomy
Heterozygous trisomy aab
Heterozygous trisomy abb
MRS locations

G

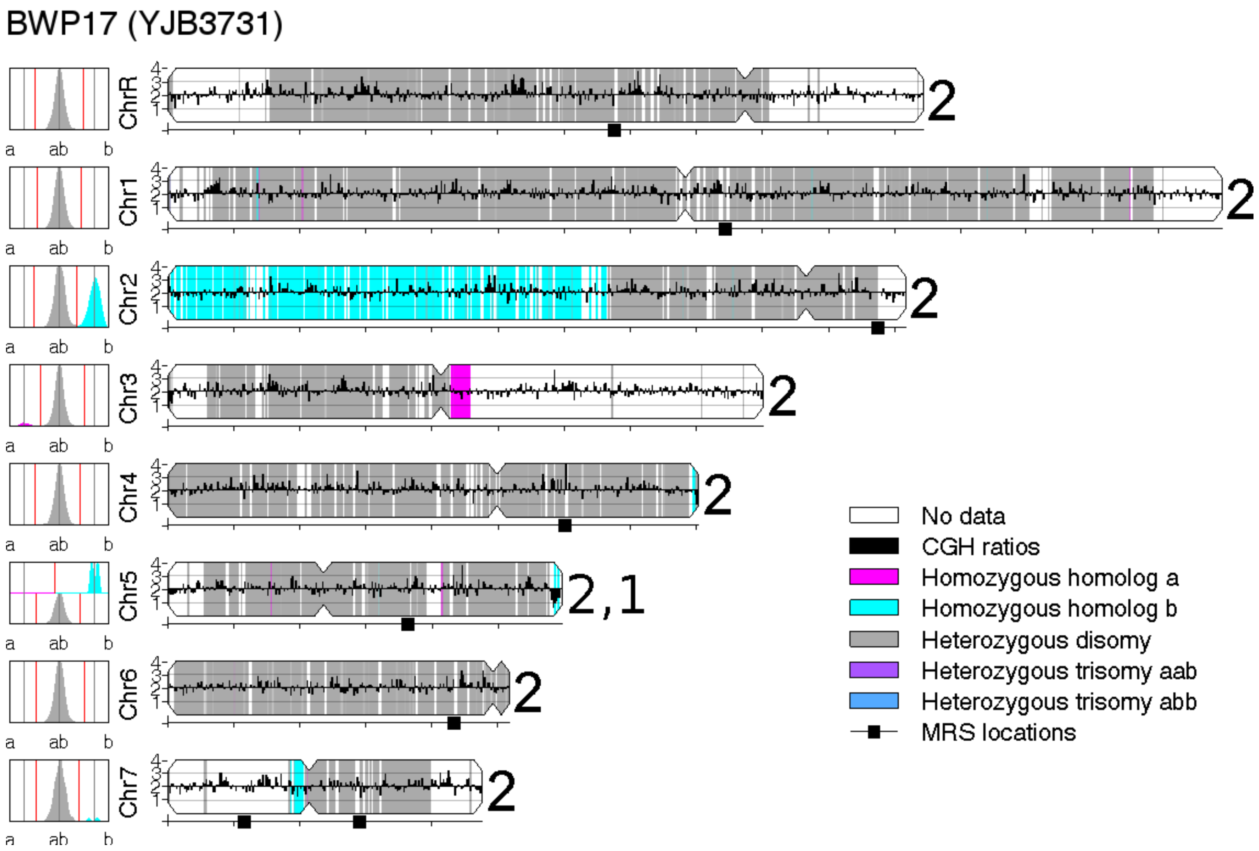

Figure S2 SNP/CGH figures of related laboratory strains derived from SC5314. Data for each strain was analyzed by SNP/CGH array and visualized as illustrated in Fig. 3. A) CAF2 (YJB2101). B) RM10 (YJB8648). C) RM100 #13 (YJB8661). D) RM1000 #2 (YJB7617). E) RM1000 #6 (YJB7616). F) SN76 (YJB10038). G) BWP17 (YJB3731).

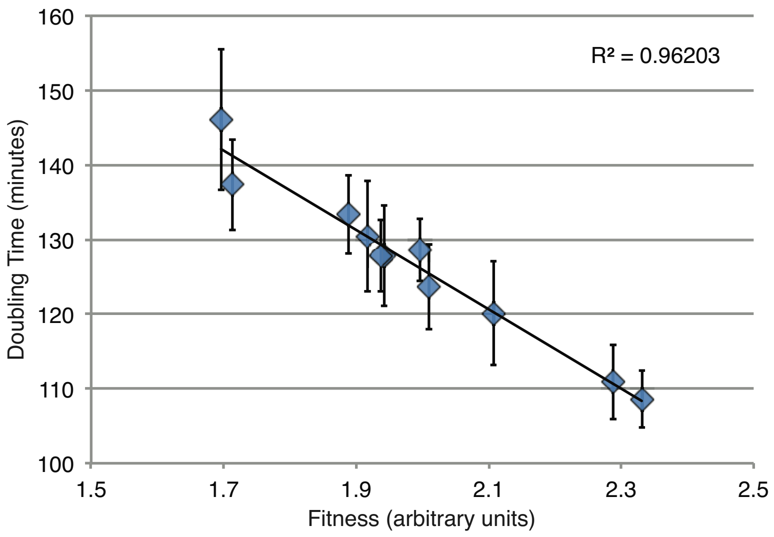

Figure S3 Doubling time correlates with strain fitness. Plot of all 10 strains analyzed in Fig. 4 for fitness (X-axis) calculated as described (Addinall *et al.* 2011) vs doubling time (Y-axis). The strong linearity indicates doubling time is the major contributor to fitness in this set of strains and growth conditions.

Addinall, S. G., E. M. Holstein, C. Lawless, M. Yu, K. Chapman *et al.*, 2011 Quantitative Fitness Analysis Shows That NMD Proteins and Many Other Protein Complexes Suppress or Enhance Distinct Telomere Cap Defects. PLoS Genet **7:** e1001362.

Tables S1-S3

Tables S1-S3 are available for download at http://www.g3journal.org/lookup/suppl/doi:10.1534/g3.111.000885/-/DC1 as excel files.

Table S1: Strains used in this study

Table S2: Informative SNPs in the complete Hapmap

Table S3: SNPs on the array that were not informative
