# Supplementary material for: High-Resolution SNP/CGH Microarrays Reveal the Accumulation of Loss of Heterozygosity in Commonly Used Candida albicans Strains
Source: G3 (Bethesda). 2011 Dec 1;1(7):523–30. doi: 10.1534/g3.111.000885 (PMC3276171; doi:10.1534/g3.111.000885)
Supplement: Supporting Information [file supp_1.7.523_FigureS1.pdf]

A

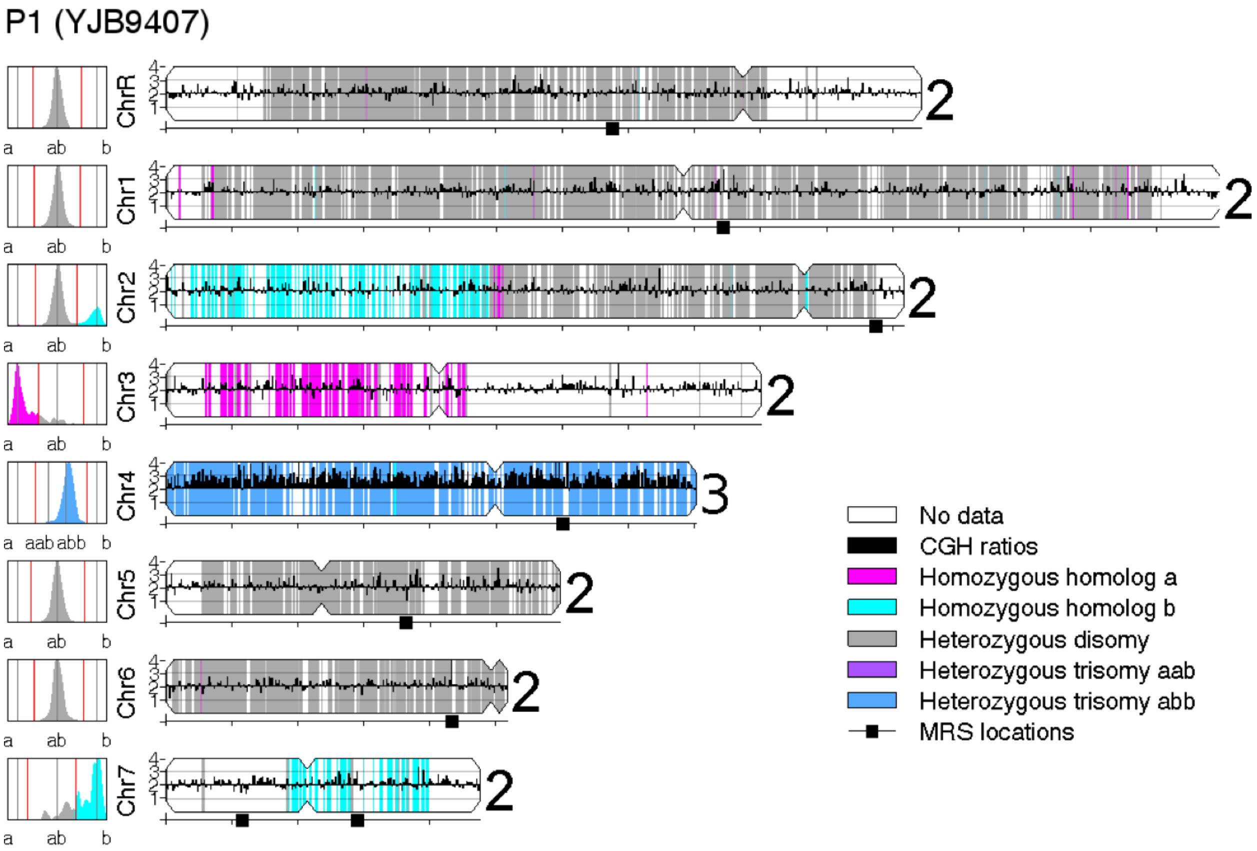
P1 (YJB9407)
ChrR
Chr1
Chr2
Chr3
Chr4
Chr5
Chr6
Chr7
a ab b
a aab abb b
2
3
No data
CGH ratios
Homozygous homolog a
Homozygous homolog b
Heterozygous disomy
Heterozygous trisomy aab
Heterozygous trisomy abb
MRS locations

B

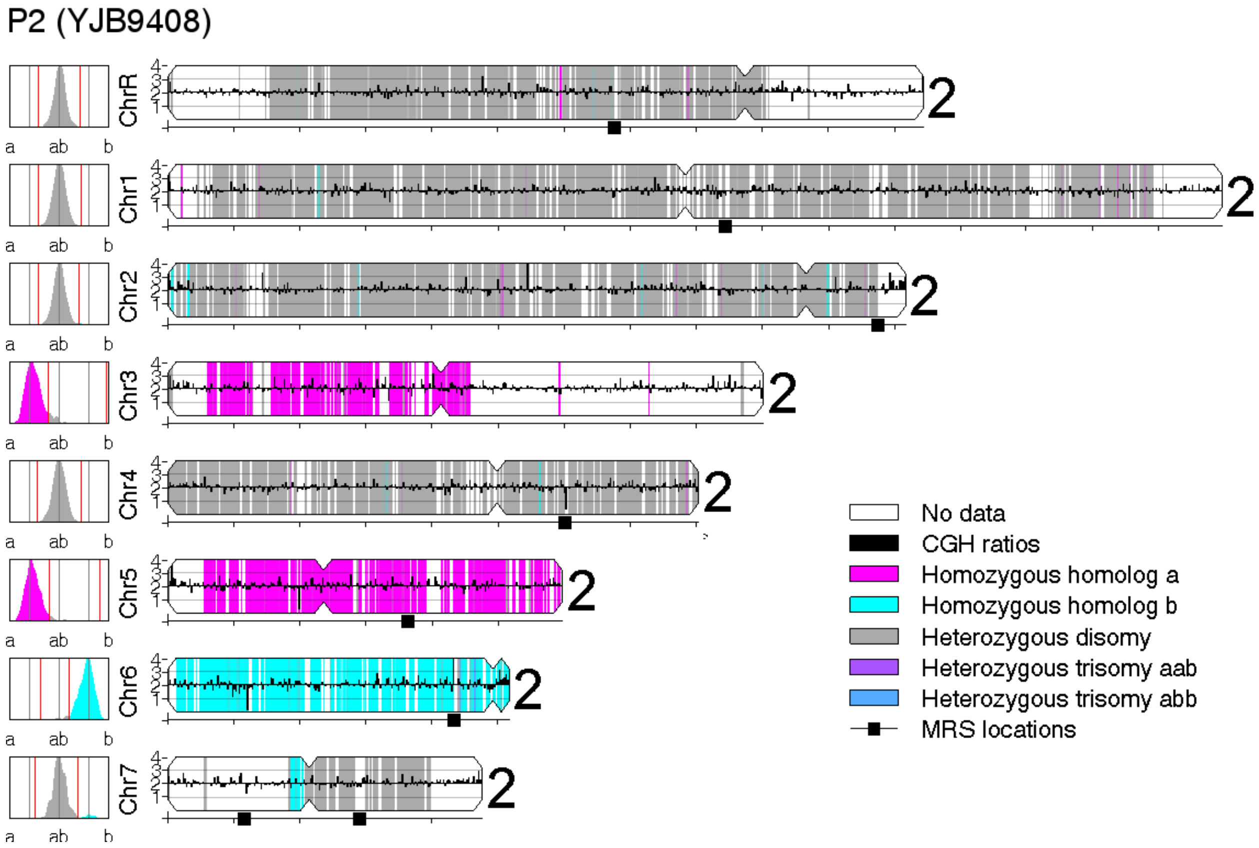
P2 (YJB9408)
ChrR
Chr1
Chr2
Chr3
Chr4
Chr5
Chr6
Chr7
a
ab
b
2
No data
CGH ratios
Homozygous homolog a
Homozygous homolog b
Heterozygous disomy
Heterozygous trisomy aab
Heterozygous trisomy abb
MRS locations

C

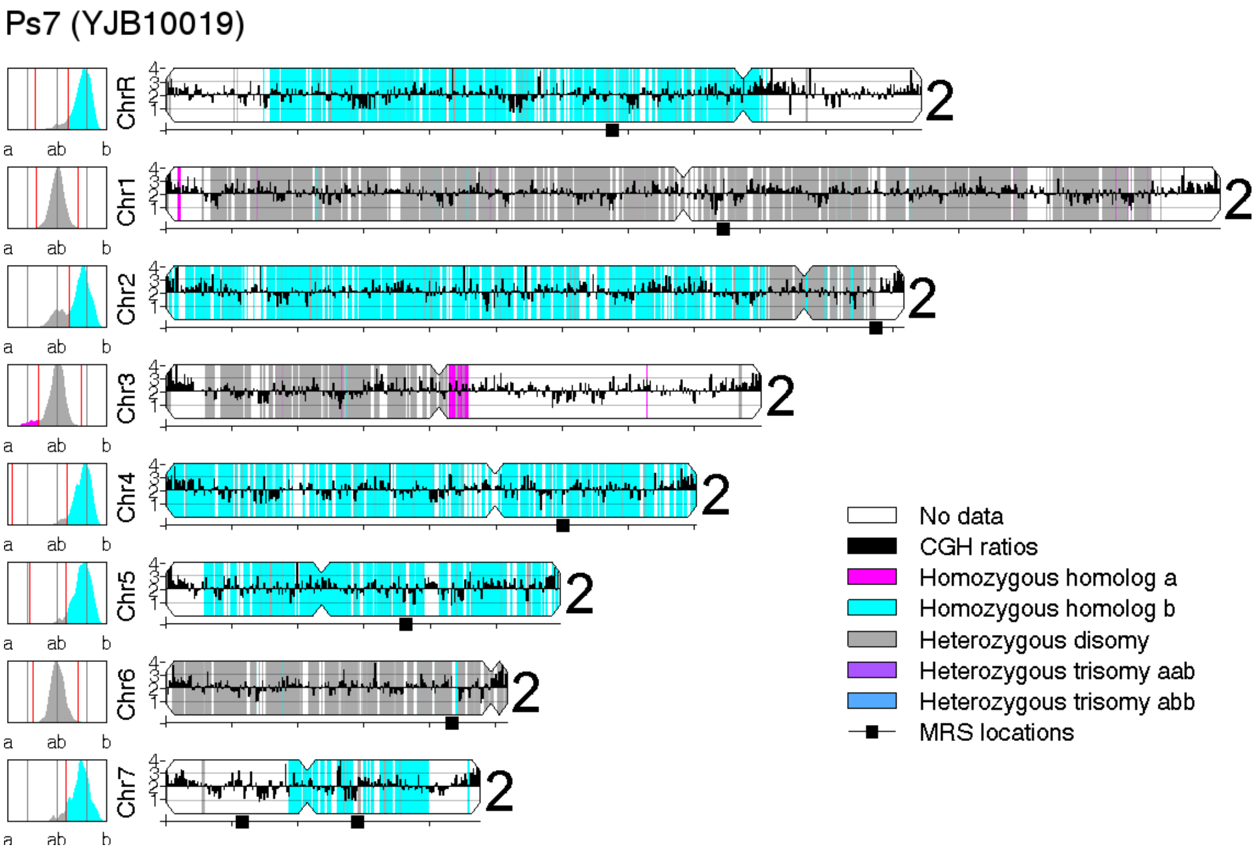

Ps7 (YJB10019)
ChrR
Chr1
Chr2
Chr3
Chr4
Chr5
Chr6
Chr7
a
ab
b
4
3
2
1
No data
CGH ratios
Homozygous homolog a
Homozygous homolog b
Heterozygous disomy
Heterozygous trisomy aab
Heterozygous trisomy abb
MRS locations

D

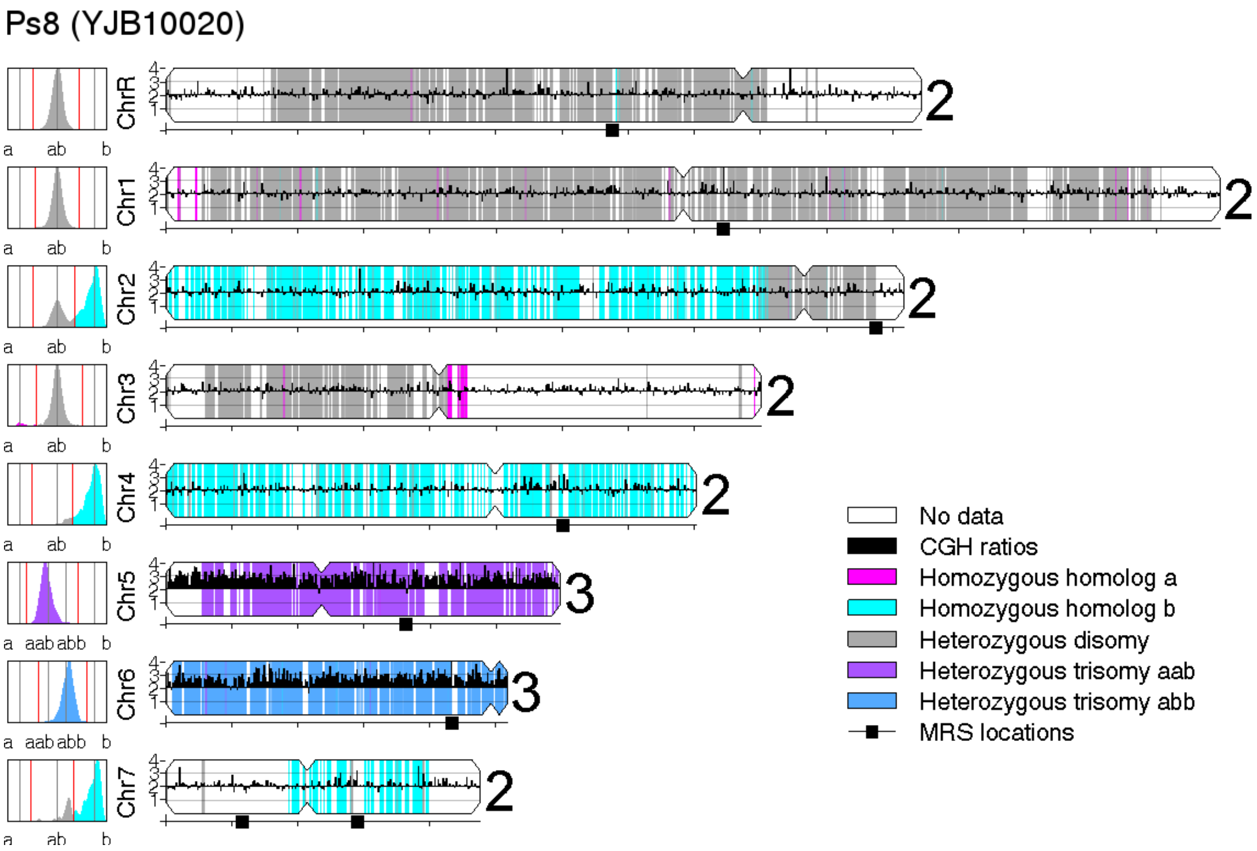
Ps8 (YJB10020)
ChrR
Chr1
Chr2
Chr3
Chr4
Chr5
Chr6
Chr7
a
ab
b
aab
abb
2
3
No data
CGH ratios
Homozygous homolog a
Homozygous homolog b
Heterozygous disomy
Heterozygous trisomy aab
Heterozygous trisomy abb
MRS locations

E

S3 (YJB9416)

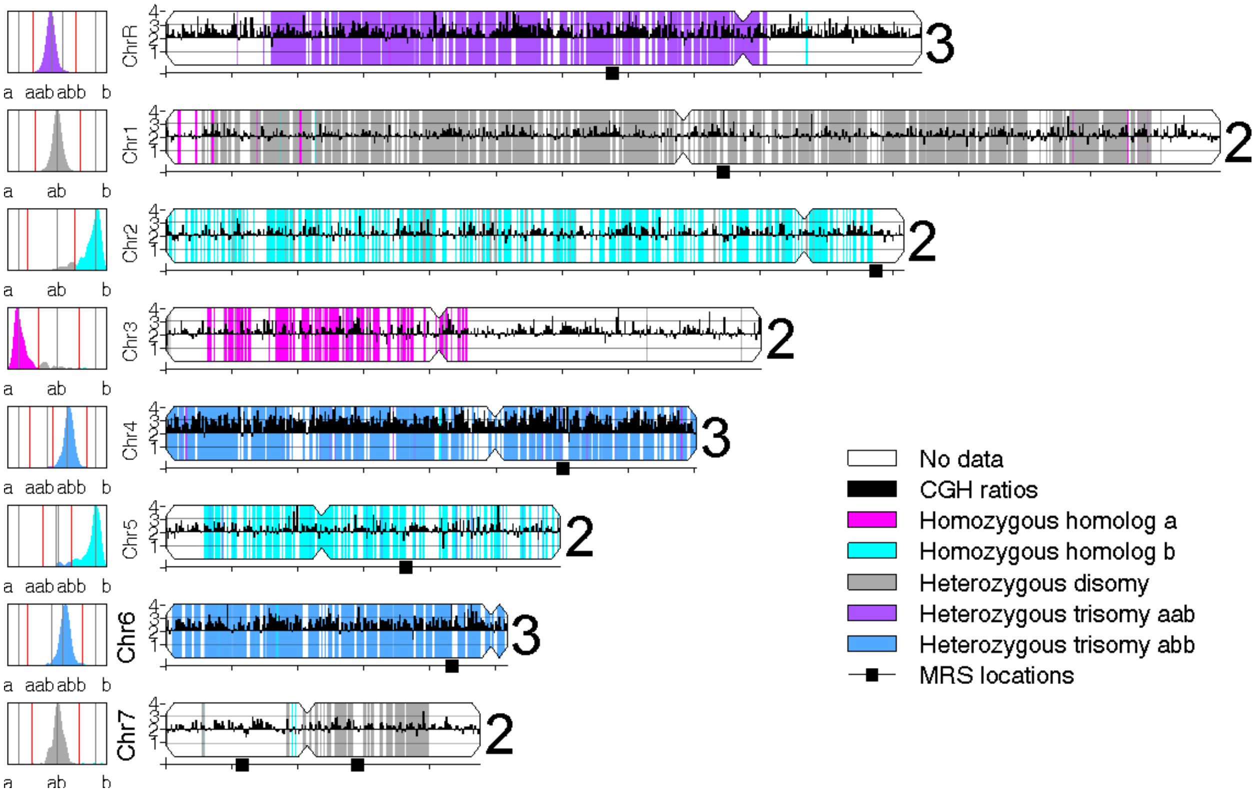
ChrR
Chr1
Chr2
Chr3
Chr4
Chr5
Chr6
Chr7
a aab abb b
a ab b
3
2
2
2
3
2
3
2
No data
CGH ratios
Homozygous homolog a
Homozygous homolog b
Heterozygous disomy
Heterozygous trisomy aab
Heterozygous trisomy abb
MRS locations

F

S4 (YJB9417)

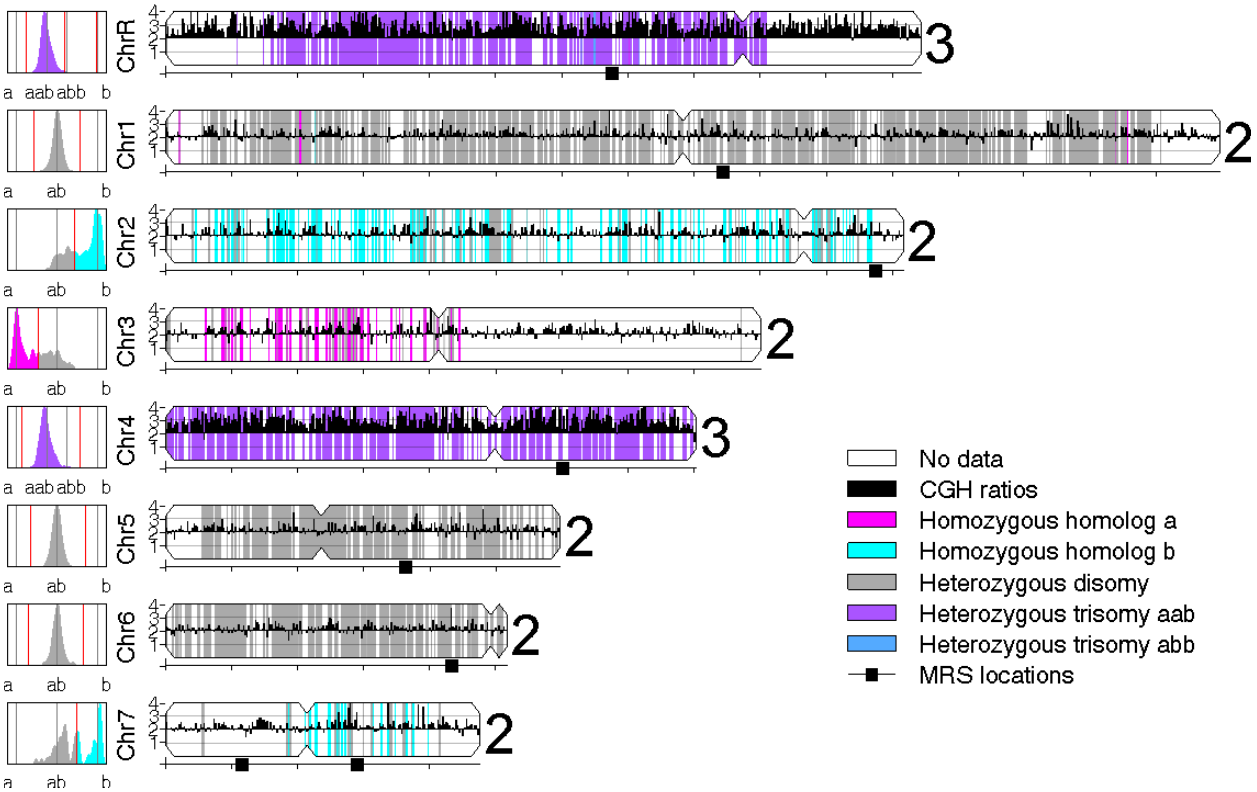

ChrR
Chr1
Chr2
Chr3
Chr4
Chr5
Chr6
Chr7
a aab abb b
a ab b
3
2
2
2
3
2
2
2
No data
CGH ratios
Homozygous homolog a
Homozygous homolog b
Heterozygous disomy
Heterozygous trisomy aab
Heterozygous trisomy abb
MRS locations

G

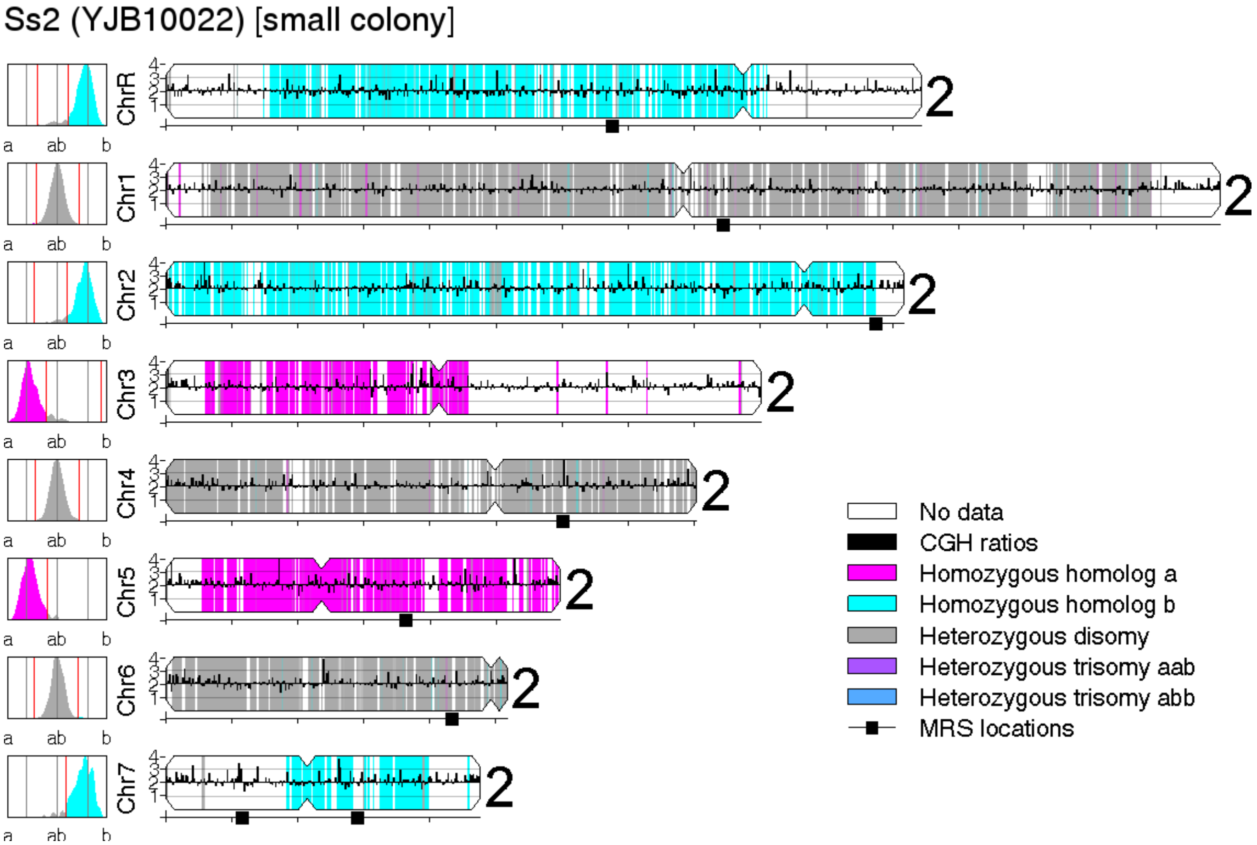
Ss2 (YJB10022) [small colony]
ChrR
Chr1
Chr2
Chr3
Chr4
Chr5
Chr6
Chr7
a
ab
b
2
No data
CGH ratios
Homozygous homolog a
Homozygous homolog b
Heterozygous disomy
Heterozygous trisomy aab
Heterozygous trisomy abb
MRS locations

H

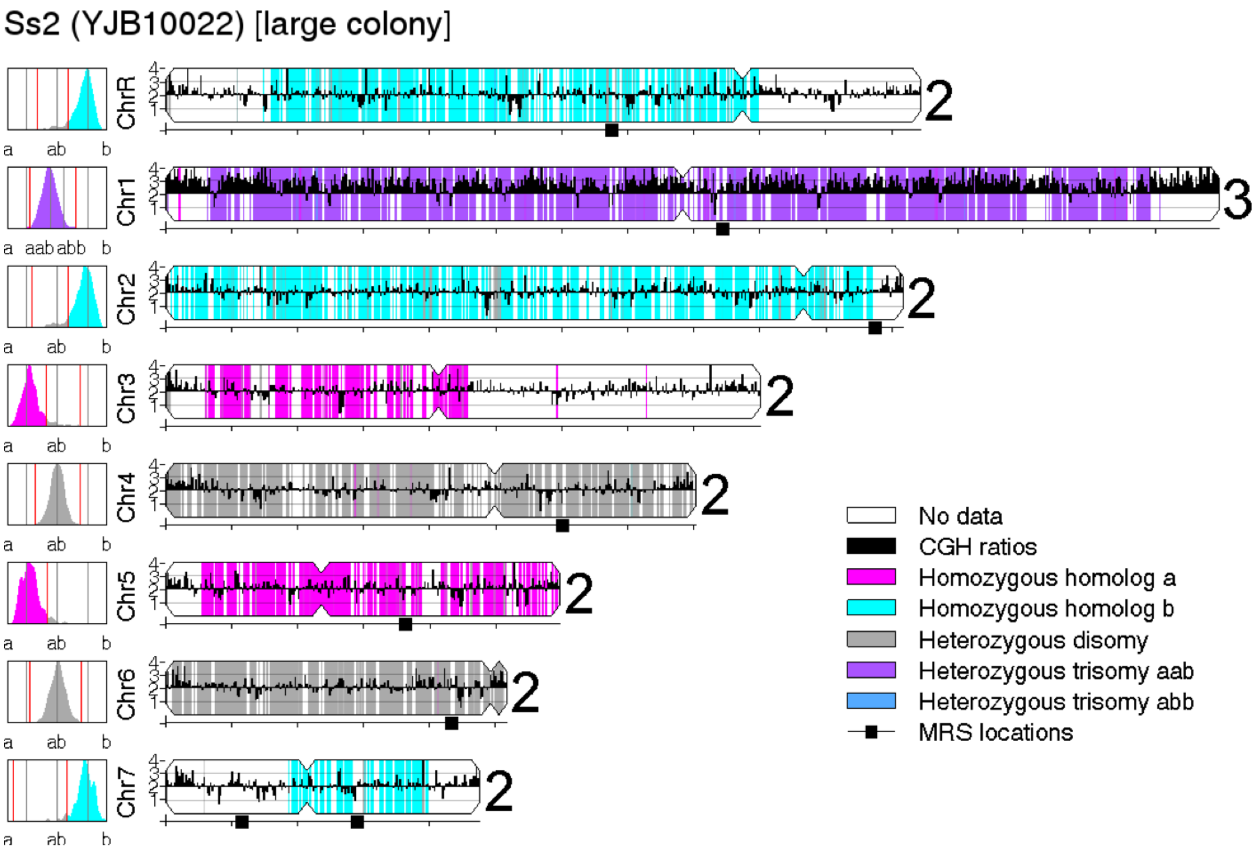

Figure S1 SNP/CGH of parasexual progeny strains used to construct the *C. albicans* SC5314 hapmap. Data for each strain was analyzed by SNP/CGH array and visualized as illustrated in Fig. 3. A) Strain P1 (YJB9407); B) P2 (YJB9408). C) Ps7 (YJB10019). D) Ps8 (YJB10020). E) S3 (YJB9416). F) S4 (YJB9417). G) Ss2 (YJB10022), small colony. H) Ss2 (YJB10022), large colony.
