# Supplementary material for: High-Resolution SNP/CGH Microarrays Reveal the Accumulation of Loss of Heterozygosity in Commonly Used Candida albicans Strains
Source: G3 (Bethesda). 2011 Dec 1;1(7):523–30. doi: 10.1534/g3.111.000885 (PMC3276171; doi:10.1534/g3.111.000885)
Supplement: Supporting Information [file supp_1.7.523_FigureS2.pdf]

A

CAF2 (YJB2101)

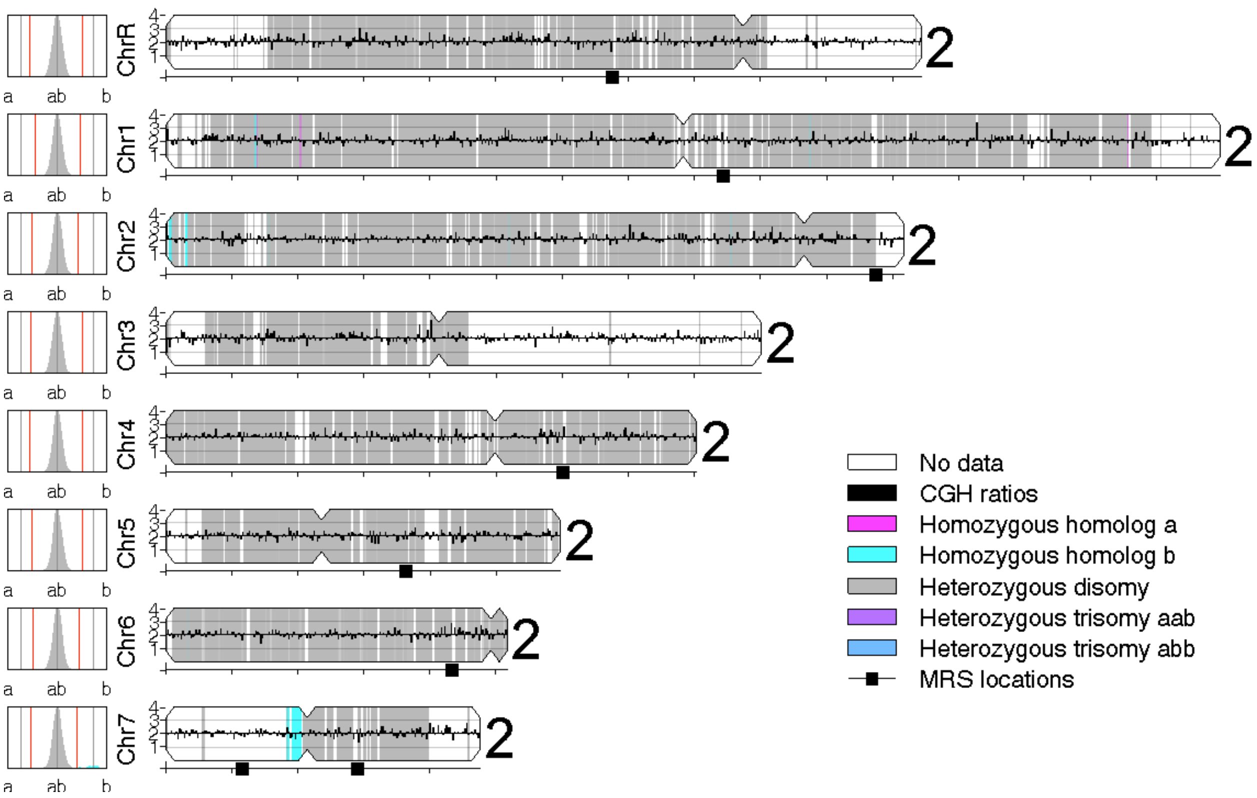

B

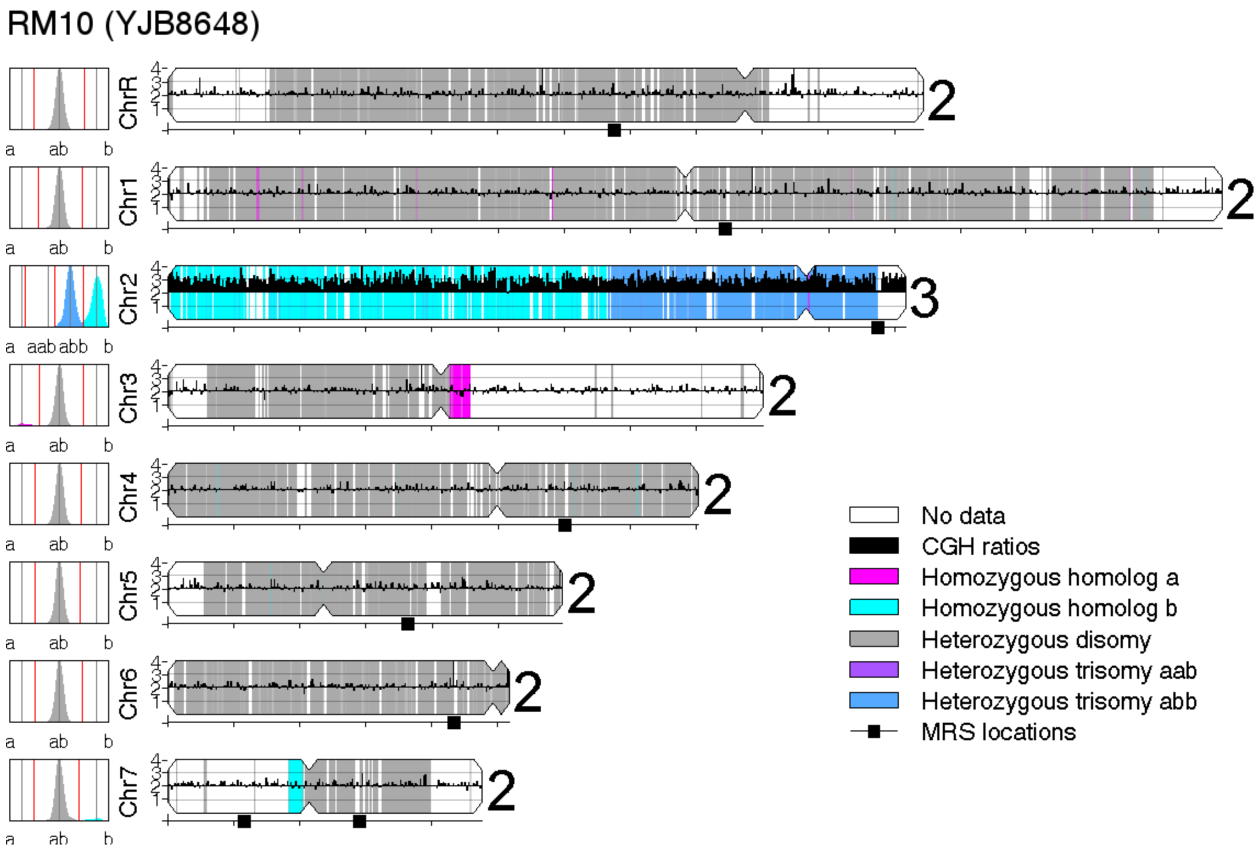
RM10 (YJB8648)
ChrR
Chr1
Chr2
Chr3
Chr4
Chr5
Chr6
Chr7
a
ab
b
aab
abb
No data
CGH ratios
Homozygous homolog a
Homozygous homolog b
Heterozygous disomy
Heterozygous trisomy aab
Heterozygous trisomy abb
MRS locations

C

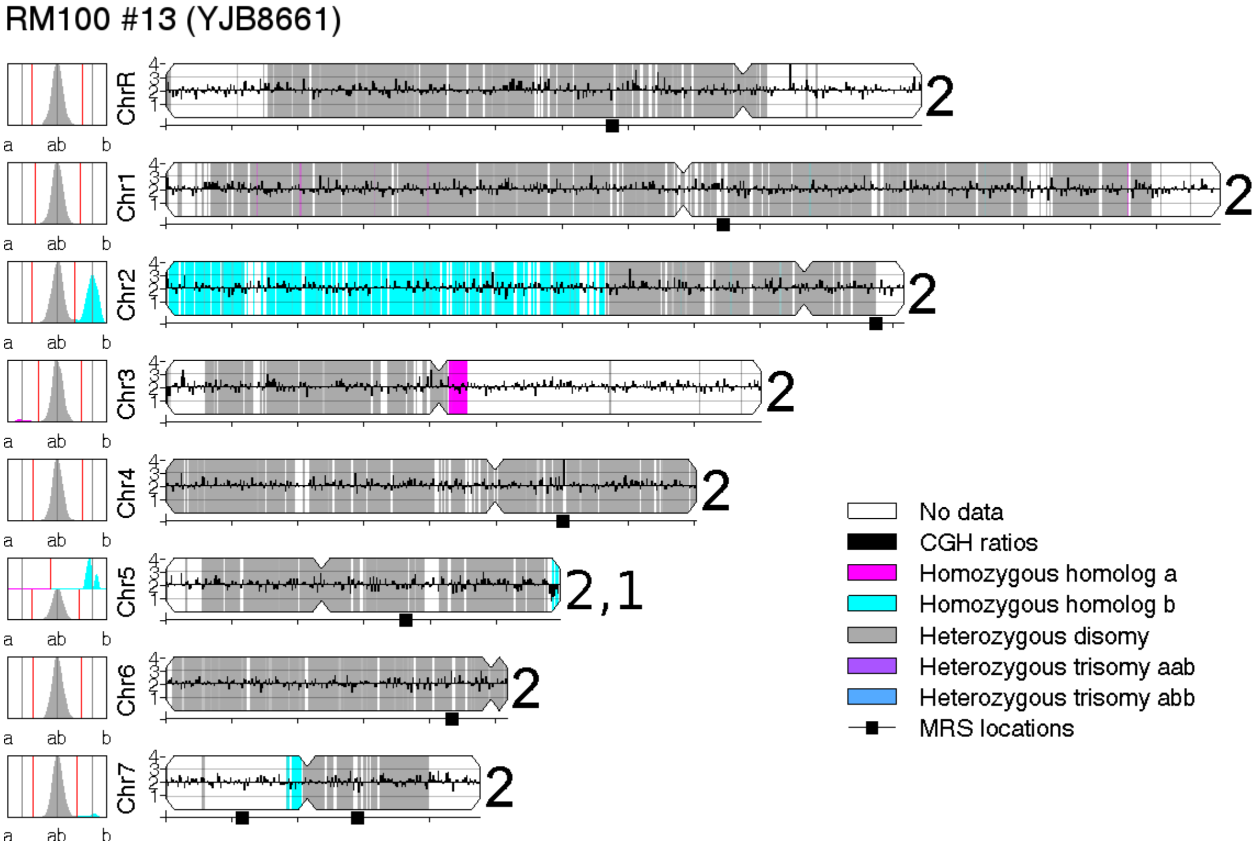
RM100 #13 (YJB8661)
ChrR
Chr1
Chr2
Chr3
Chr4
Chr5
Chr6
Chr7
a
ab
b
2
2
2
2
2
2,1
2
2
No data
CGH ratios
Homozygous homolog a
Homozygous homolog b
Heterozygous disomy
Heterozygous trisomy aab
Heterozygous trisomy abb
MRS locations

D

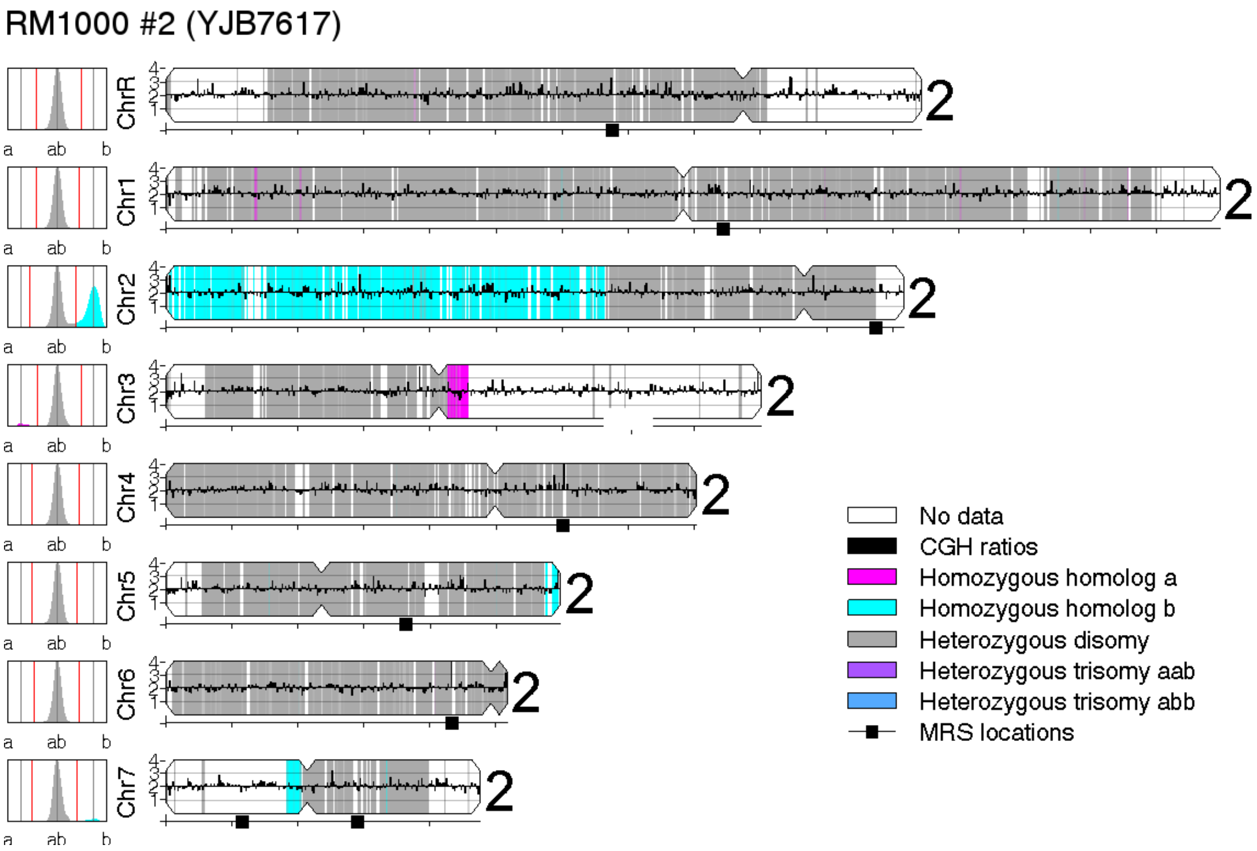
RM1000 #2 (YJB7617)
ChrR
Chr1
Chr2
Chr3
Chr4
Chr5
Chr6
Chr7
a
ab
b
2
No data
CGH ratios
Homozygous homolog a
Homozygous homolog b
Heterozygous disomy
Heterozygous trisomy aab
Heterozygous trisomy abb
MRS locations

E

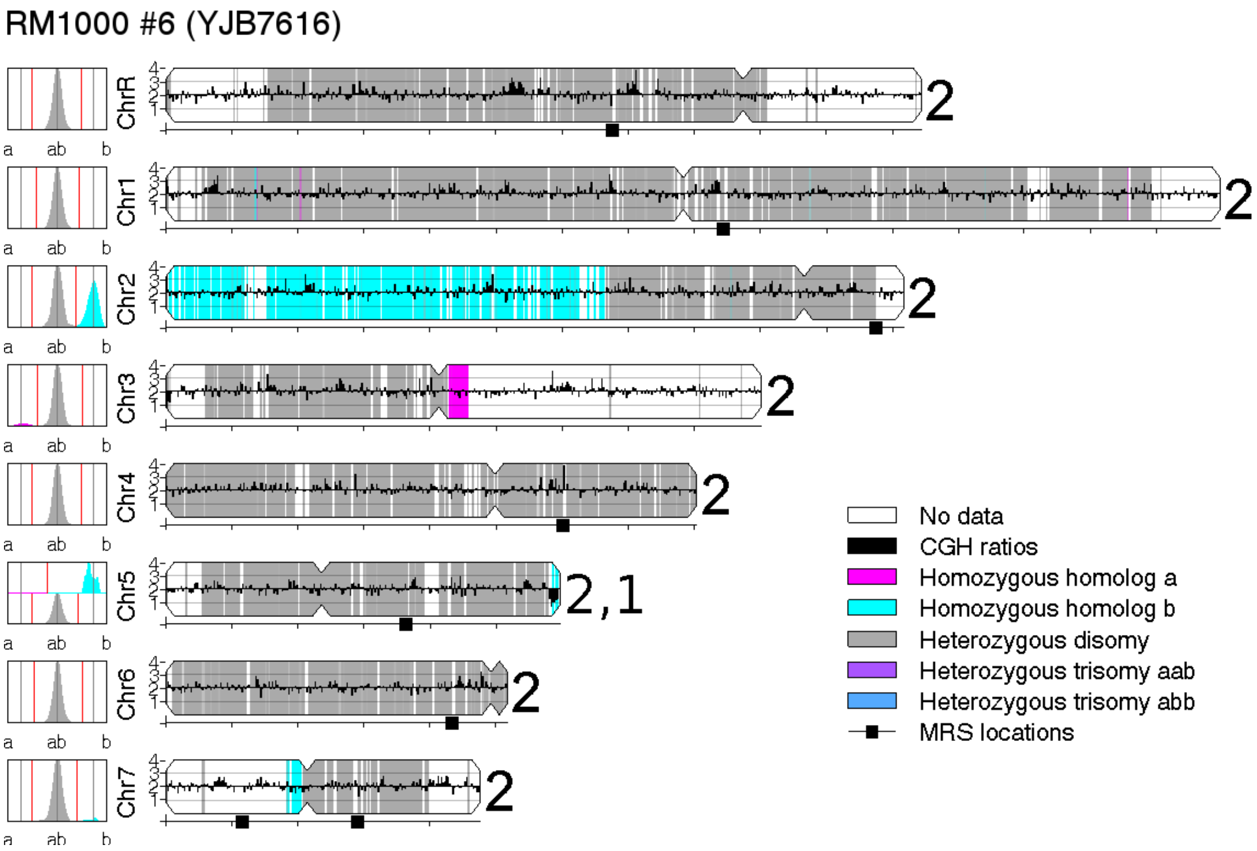

RM1000 #6 (YJB7616)
ChrR
Chr1
Chr2
Chr3
Chr4
Chr5
Chr6
Chr7
a
ab
b
2
2,1
No data
CGH ratios
Homozygous homolog a
Homozygous homolog b
Heterozygous disomy
Heterozygous trisomy aab
Heterozygous trisomy abb
MRS locations

F

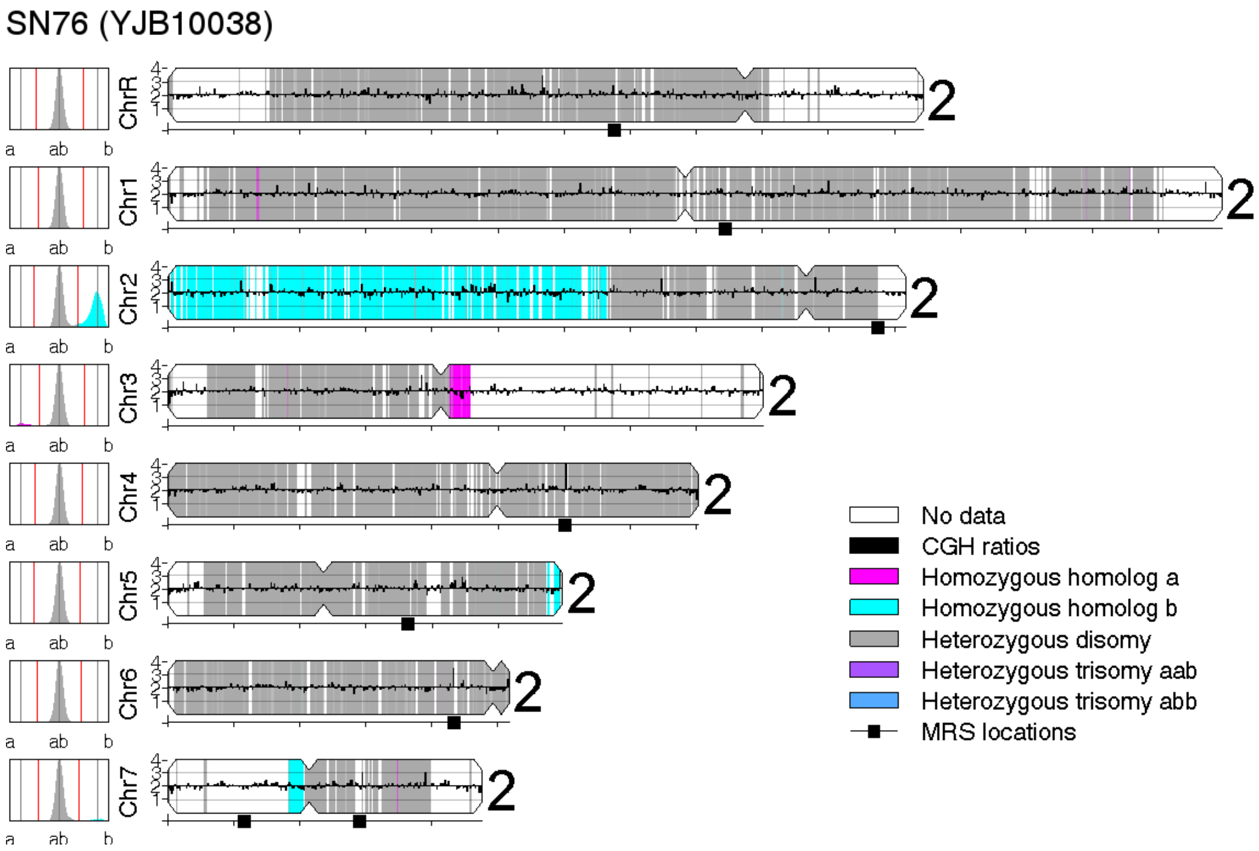
SN76 (YJB10038)
ChrR
Chr1
Chr2
Chr3
Chr4
Chr5
Chr6
Chr7
a
ab
b
1
2
3
4
No data
CGH ratios
Homozygous homolog a
Homozygous homolog b
Heterozygous disomy
Heterozygous trisomy aab
Heterozygous trisomy abb
MRS locations

G

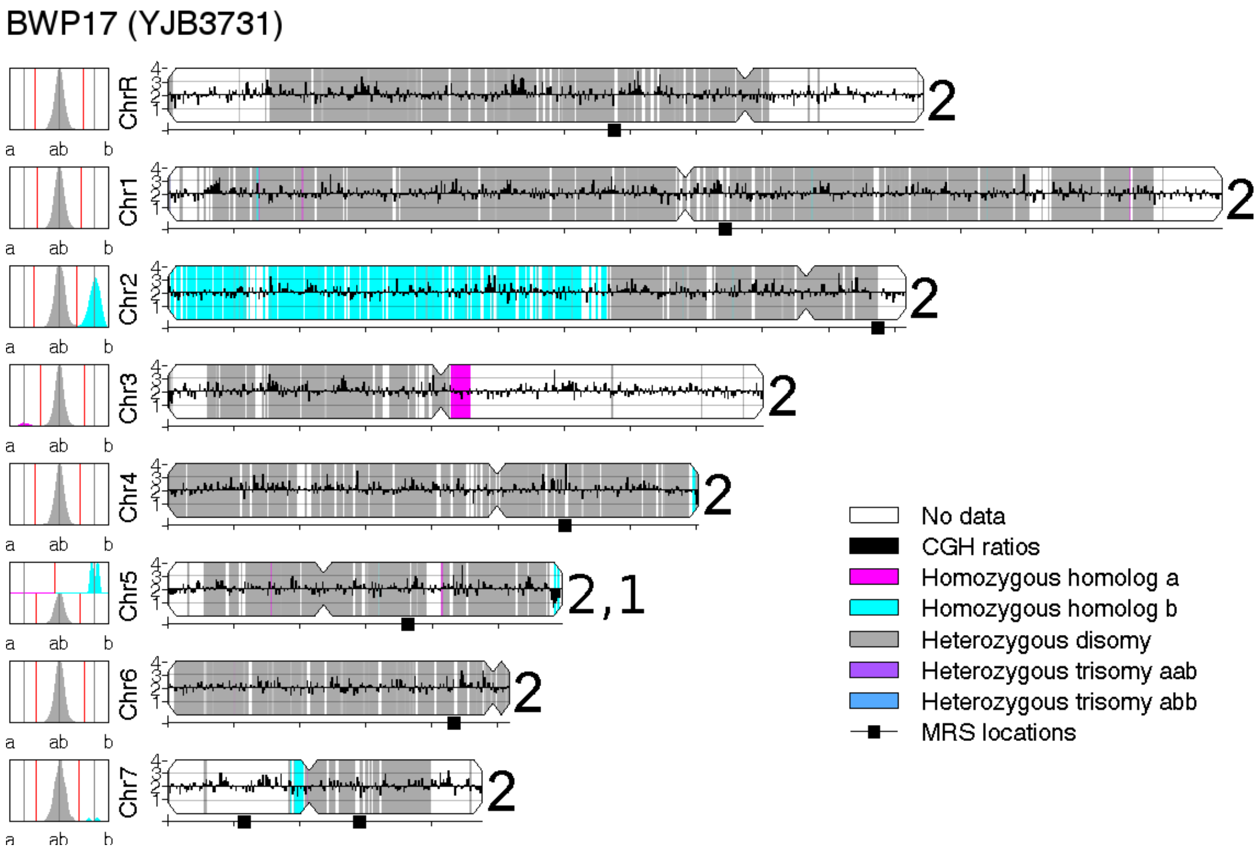

Figure S2 SNP/CGH figures of related laboratory strains derived from SC5314. Data for each strain was analyzed by SNP/CGH array and visualized as illustrated in Fig. 3. A) CAF2 (YJB2101). B) RM10 (YJB8648). C) RM100 #13 (YJB8661). D) RM1000 #2 (YJB7617). E) RM1000 #6 (YJB7616). F) SN76 (YJB10038). G) BWP17 (YJB3731).
