# Supplementary material for: High-Resolution SNP/CGH Microarrays Reveal the Accumulation of Loss of Heterozygosity in Commonly Used Candida albicans Strains
Source: G3 (Bethesda). 2011 Dec 1;1(7):523–30. doi: 10.1534/g3.111.000885 (PMC3276171; doi:10.1534/g3.111.000885)
Supplement: Supporting Information [file supp_1.7.523_FigureS3.pdf]

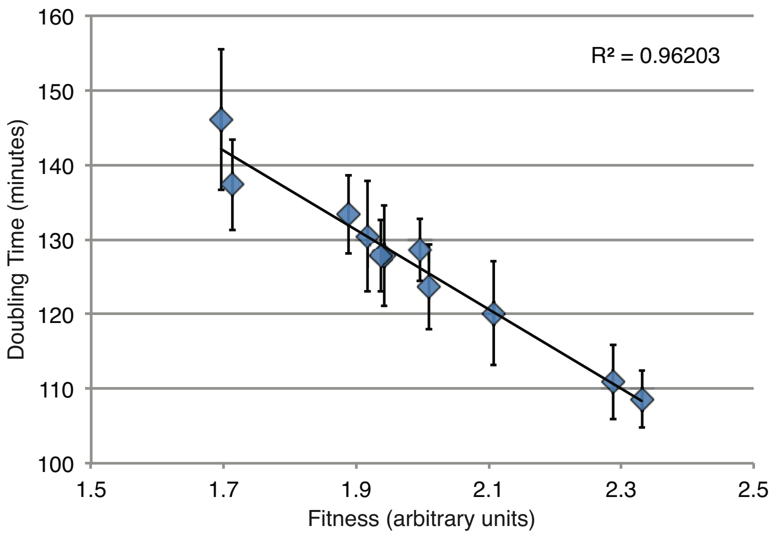

Figure S3 Doubling time correlates with strain fitness. Plot of all 10 strains analyzed in Fig. 4 for fitness (X-axis) calculated as described (ADDINALL *et al.* 2011) vs doubling time (Y-axis). The strong linearity indicates doubling time is the major contributor to fitness in this set of strains and growth conditions.

ADDINALL, S. G., E. M. HOLSTEIN, C. LAWLESS, M. YU, K. CHAPMAN *et al.*, 2011 Quantitative Fitness Analysis Shows That NMD Proteins and Many Other Protein Complexes Suppress or Enhance Distinct Telomere Cap Defects. PLoS Genet **7:** e1001362.
