# Supplementary material for: High-Resolution SNP/CGH Microarrays Reveal the Accumulation of Loss of Heterozygosity in Commonly Used Candida albicans Strains
Source: G3 (Bethesda). 2011 Dec 1;1(7):523–30. doi: 10.1534/g3.111.000885 (PMC3276171; doi:10.1534/g3.111.000885)
Supplement: Corrigendum [file supp_1_7_523_v2_index.html]

Corrigendum 

# High-Resolution SNP/CGH Microarrays Reveal the Accumulation of Loss of Heterozygosity in Commonly Used *Candida albicans* Strains

## Corrigendum for Abbey et al. G3 1 (7) 523-530.

**Files in this Data Supplement:**

- Corrigendum - In the article by D. ABBEY, M. HICKMAN, D. GRESHAM, and J. BERMAN (G3 1: 523-530) entitled "High-Resolution SNP/CGH Microarrays Reveal the Accumulation of Loss of Heterozygosity in Commonly Used Candida albicans Strains", an updated version of Table S2 of the supporting information (Informative SNPs in the complete Hapmap) is now available at http://www.g3journal.org/lookup/suppl/doi:10.1534/g3.111.000885/-/DC1/TableS2.xls.
