# Supplementary material for: High-Resolution SNP/CGH Microarrays Reveal the Accumulation of Loss of Heterozygosity in Commonly Used Candida albicans Strains
Source: G3 (Bethesda). 2011 Dec 1;1(7):523–30. doi: 10.1534/g3.111.000885 (PMC3276171; doi:10.1534/g3.111.000885)
Supplement: Corrigendum [file supp_1.7.523_Corrigendum_Abbey.pdf]

Corrigendum for Abbey *et al.* *G3* 1 (7) 523-530.

G3, Vol. 1, 523-530, December 2011, Copyright © 2011

CORRIGENDUM

In the article by D. ABBEY, M. HICKMAN, D. GRESHAM, and J. BERMAN (*G3* 1: 523-530) entitled "High-Resolution SNP/CGH Microarrays Reveal the Accumulation of Loss of Heterozygosity in Commonly Used *Candida albicans* Strains", an updated version of Table S2 of the supporting information (Informative SNPs in the complete Hapmap) is now available at http://www.g3journal.org/lookup/suppl/doi:10.1534/g3.111.000885/-/DC1/TableS2.xls.
